# Traffic Patterns of the Migrating Endothelium: How Force Transmission Regulates Vascular Malformation and Functional Shunting During Angiogenic Remodelling

**DOI:** 10.3389/fcell.2022.840066

**Published:** 2022-05-19

**Authors:** Lowell T. Edgar, Hyojin Park, Jessica R. Crawshaw, James M. Osborne, Anne Eichmann, Miguel O. Bernabeu

**Affiliations:** ^1^ Centre for Medical Informatics, Usher Institute, The University of Edinburgh, Edinburgh, United Kingdom; ^2^ Cardiovascular Research Center Department of Internal Medicine, Yale University School of Medicine, New Haven, CT, United States; ^3^ School of Mathematics and Statistics, University of Melbourne, Melbourne, VIC, Australia; ^4^ Yale University School of Medicine, Department of Cellular and Molecular Physiology, New Haven, CT, United States; ^5^ Université de Paris, PARCC, INSERM, Paris, France; ^6^ The Bayes Centre, The University of Edinburgh, Edinburgh, United Kingdom

**Keywords:** angiogenesis, angiogenic remodelling, endothelial cells, collective migration, flow-migration coupling, force transmission, migration dynamics, arteriovenous malformations

## Abstract

Angiogenesis occurs in distinct phases: initial spouting is followed by remodelling in which endothelial cells (ECs) composing blood vessels rearrange by migrating against the direction of flow. Abnormal remodelling can result in vascular malformation. Such is the case in mutation of the Alk1 receptor within the mouse retina which disrupts flow-migration coupling, creating mixed populations of ECs polarised with/against flow which aggregate into arteriovenous malformations (AVMs). The lack of live imaging options *in vivo* means that the collective EC dynamics that drive AVM and the consequences of mixed populations of polarity remain a mystery. Therefore, our goal is to present a novel agent-based model to provide theoretical insight into EC force transmission and collective dynamics during angiogenic remodelling. Force transmission between neighbouring agents consists of extrusive forces which maintain spacing and cohesive forces which maintain the collective. We performed migration simulations within uniformly polarised populations (against flow) and mixed polarity (with/against flow). Within uniformly polarised populations, extrusive forces stabilised the plexus by facilitating EC intercalation which ensures that cells remained evenly distributed. Excess cohesion disrupts intercalation, resulting in aggregations of cells and functional shunting. Excess cohesion between ECs prevents them from resolving diameter balances within the plexus, leading to prolonged flow reversals which exert a critical behaviour change within the system as they switch the direction of cell migration and traffic patterns at bifurcations. Introducing mixtures of cell polarity dramatically changed the role of extrusive forces within the system. At low extrusion, opposing ECs were able to move past each other; however, at high extrusion the pushing between cells resulted in migration speeds close to zero, forming traffic jams and disrupting migration. In our study, we produced vascular malformations and functional shunting with either excess cohesion between ECs or mixtures of cell polarity. At the centre of both these mechanisms are cell-cell adherens junctions, which are involved in flow sensing/polarity and must remodelling dynamically to allow rearrangements of cells during vascular patterning. Thus, our findings implicate junctional dysfunction as a new target in the treatment and prevention of vascular disease and AVMs.

## Introduction

Angiogenesis occurs in two distinct phases: an initial sprouting phase which establishes the primitive plexus, and a remodelling phase in which the vasculature rearranges based on signals received from blood flow. Although sprouting angiogenesis has been extensively studied for many years now, angiogenic remodelling has received much less attention in comparison. The primary driver of remodelling is endothelial cells (ECs) responding to shear stress along the lumen wall by polarising and migrating against the direction of flow, generally from regions of low shear to high (Franco et al., 2015; Georgieva et al., 2019). A vascular plexus can be considered as a stable coexistence of long low-shear distal pathways with shorter high-shear arteriovenous connections (Pries et al., 2010). Pries et al. and others have historically demonstrated that vascular adaptation and remodelling governed by shear stress alone creates “the shunt problem” in which cells prioritise short arteriovenous pathways at the expense of longer distal pathways, thereby shunting both cells and blood flow away from vital regions of tissue (Hacking et al., 1996; Pries et al., 1998; Pries et al., 2010; Edgar et al., 2021). Arteriovenous shunts (also referred to as arteriovenous malformations, or AVMs) can pathologically arise in numerous tissues including the liver (Copel et al., 2018), coronary circulation (Gatzoulis et al., 2018), lungs (Cartin-Ceba et al., 2013), and brain (Principles of Neurological Surgery, 2012), often with detrimental effects on health and mortality. In particular, AVMs in the brain are an important cause of haemorrhagic stroke, and surgical treatment options are invasive and limited (Derdeyn et al., 2017; Di Bartolomeo et al., 2021). The lack of arteriovenous shunts in healthy vasculature suggests there must be additional mechanisms beyond shear stress which regulate remodelling and prevent shunting, as originally proposed by (Pries et al., 2010). However, no unequivocal evidence of these mechanisms has been presented to date, and the exact mechanisms which promote or prevent shunt formation during vascular remodelling remain a mystery.

The activin receptor-like kinase 1 (Alk1) is an endothelial transmembrane receptor for ligands within the transforming growth factor- β (Tgfβ) superfamily and plays a crucial role in vascular development and disease (Roman and Hinck, 2017). Mutation in Alk1 results in a vascular disorder known as hereditary haemorrhagic telangiectasia (HHT, specifically HHT2 in the case of Alk1 mutation) in which patients are afflicted with AVMs throughout the body (Shovlin, 2010). It has recently been shown that induced deletion of Alk1 in capillary-venous ECs is sufficient to induce AVMs in the postnatal mouse retina (a common *in vivo* model of developmental angiogenesis) (Park et al., 2021). Interestingly, ECs in these Alk1 deficient mice appear to have lost proper flow-migration coupling and present as mixed populations of cells aligned either with or against blood flow. Although these AVM-producing mice present an exciting new platform for which to investigate the mechanisms governing AVM formation, the lack of live-imaging options in the postnatal mouse retina means we have little information on the collective dynamics of ECs during angiogenic remodelling, and even less on the full implications of mixed polarity populations during AVM formation. Therefore, our goal in this study is to present a novel agent-based model (ABM) for which we can supplement existing experimental efforts with theoretical insight into EC collective dynamics during angiogenic remodelling in both uniform and mixed polarity cell populations. With this model, we will demonstrate that the factors that control force transmission between individual migrating ECs exert powerful control over the remodelling outcome, capable of pushing the system from a stable vascular plexus towards formation of AVMs. Further, introducing mixed polarity populations (both ECs aligned with and against the flow) has a dramatic impact on collective migration and changes the very nature of force transmission between ECs in which factors that normally stabilise the plexus instead create traffic jams within the collective and promote vascular defects and malformation. Taken together, our findings demonstrate the crucial role of force transmission within the migrating endothelium as an important regulator of vascular development which can either prevent or promote AVM formation during angiogenic remodelling.

## Results

### AVMs Consist of Aggregates of ECs With Mixed Polarity With/Against Flow

**FIGURE 1:**
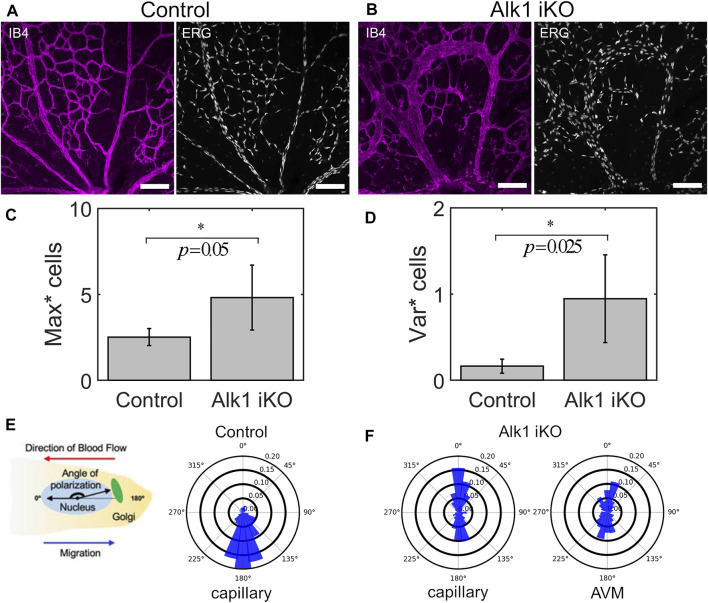
AVMs in Alk1 deficient mice consist of aggregates of ECs with mixed polarity with/against flow. **(A)** P6 retinal vasculature from Control mice show an even disruption of ECs throughout the capillary plexus through imaging of the endothelial lumen (IB4) and individual cell nuclei (ERG) (scale bar 100 μm). **(B)** Induced knockout of Alk1 signalling *via* tamoxifen injection at P4 in *Alk1* ^*f/f*^ *Mfsd2a Cre* ^*ERT2*^ mice produces pronounced AVMs by P6. AVMs feature enlarged lumens consisting of aggregates of numerous cells. **(C)** By assigning cell nuclei to vessels, we find that the maximum number of cells amongst vessels is significantly higher in Alk1 deficient mice compared to Controls, demonstrating cell aggregation within AVMs. Statistical significance between measurements in Alk1 iKO plexuses vs. Control was assessed *via* Welch’s *t*-test (α = 0.05) **(D)** Similarly, the variance in cell number amongst vessels is significantly higher in Alk1 deficient mice compared to Controls, demonstrating the uneven distribution of cells with capillary plexuses featuring AVMs. **(E)** Nucleus-to-Golgi polarity was measured in individual ECs and expressed with respect to the direction of blood flow predicted *via* computational modelling. In Control mice, distributions of polarity angles show that cells within the capillary plexus are highly aligned against the direction of blood flow at P6. **(F)** In Alk1 deficient mice, we found mixed polarity with/against flow amongst cells within the capillary plexus. In particular, we found that AVMs consist of near 50/50 mix of polarity with/against flow.

Induced deletion of Alk1 amongst capillary-venous ECs in *Alk1* ^*f/f*^ *Mfsd2a Cre* ^*ERT2*^ mutant mice (hence referred to as Alk1 iKO) results in characteristic retinal AVMs (Figures 1A,B). Briefly, Alk1 was deleted at P4 and retinal vasculature was dissected out and fixed at P6. The endothelial lumen was fluorescently labelled *via* IB4 and EC nuclei *via* ERG. After confocal imaging, we skeletonised capillary plexuses labelled for IB4 in both Control and Alk1 iKO mice into a graph (i.e., collection of nodes and edges), and assigned each ERG labelled EC nuclei to the nearest vessel edge (with edge being defined as a portion of vessel between bifurcation points or end points). Raw image data and retinal wedge graphs for each Control and Alk1 iKO mouse can be found in Supplementary Figures S1, 2, respectively. We found that AVMs in Alk1 iKO mice consisted of a large number of cells aggregated along a single flow path, and an increase in the maximum number of cells amongst vessels compared to Control mice (Figure 1C). ECs within capillary plexuses of Control mice were also more evenly distributed compared to the wider distribution in Alk1 iKO mice, as seen *via* an increase in the variance in cell number amongst vessels (Figure 1D). Taken together, these results demonstrate that AVMs in Alk1 iKO mice consist of large aggregations of ECs and a skewed distribution of ECs relative to Controls. Measurements of Nucleus-to-Golgi polarity were taken relative to the direction of blood flow calculated by computational simulations. In Control mice, capillary ECs were found to be predominately polarized against the direction of blood flow (Figure 1E). However, capillary ECs in Alk1 iKO mice had a mixture of polarities with/against flow (Figure 1F). ECs within the AVMs consisted of a similar mixture of polarities with/against flow. These experimental findings pose two major questions that we will use as the basis for our study: 1) what changes in force transmission between migrating ECs cause cells to aggregate and distribute unevenly along a minority of flow pathways as seen in AVMs? And 2), what are the effects of introducing mixtures of cell polarity on EC collective dynamics and angiogenic remodelling? Due to insufficient means of live imaging EC dynamics and inference of forces transmitted between cells in the *in vivo* mouse retina, we aim to design a novel force-driven ABM of flow-migration coupling to elucidate and demonstrate the important role of force transmission between ECs during angiogenic remodelling and the creation of AVMs.

### The Migrating Endothelium as Overlapping Ellipses

**FIGURE 2:**
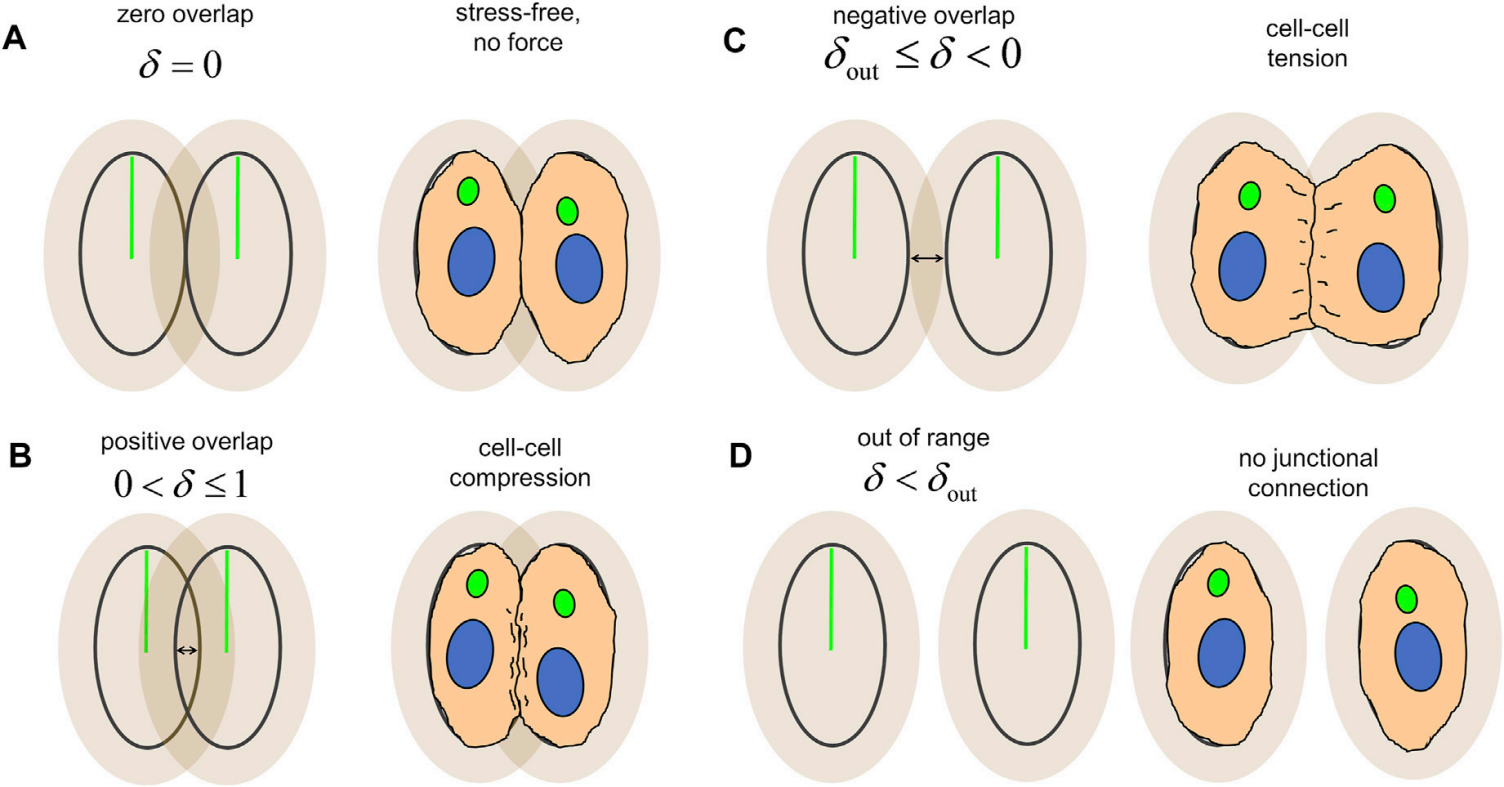
Overlap between agents, δ, produces force transmission and serves as an analogy for cellular mechanics. **(A)** Agents adjacent to each other at the boundary of the inner ellipse are in the “stress-free” configuration and transmit no force between one another. **(B)** Overlap of the inner ellipses produce an extrusive force which pushes the cells apart and represents cell-cell compression. **(C)** Overlap of the outer ellipses produces a cohesive force and which pulls the cells together and represents cell-cell tension. **(D)** Agents without overlap between their inner and outer ellipses are out of range from each other and transmit no force, indicating a lack of junctional connection between the two.

ECs were represented as agents consisting of nested polarised ellipses. The inner ellipse of each agent represents the undeformed “stress-free” state of the cell. The outer ellipse, a scaling of the inner ellipse by a factor Γ>1 , represents the “yield surface” of the cell’s junctions connections as it experiences tensile stretch, with Γ the junctional yield stretch. ECs experiencing tension at their junctions will deform along any direction up to a stretch ratio of Γ, after which they will release the junctional connection and attempt to return to the stress-free configuration. ECs transmit force to one another depending on the overlap, δ , of adjacent ellipses. As such, overlap in this model serves as an analogy for cellular mechanics (Figure 2). Overlap between two agents is defined *via* the distance between them as well as the angle at which they intersect (see Methods). Agents with inner ellipses adjacent to each other but not overlapping are defined to have no overlap (i.e., δ=0 ), do not transmit force to one another and thereby represents the stress-free configuration (Figure 2A). Agents with overlapping inner ellipses are defined to have positive overlap (ranging 0<δ≤1 ) and transmit an *extrusive force* between each other which pushes the cells back to the stress-free configuration; as such, positive overlap between agents represents a state of cell-cell compression (Figure 2B). Agents with overlapping outer ellipses are defined to have negative overlap (ranging from $\delta_{\text{out}}\leq \delta <0$ ) and transmit a *cohesive force* between each other which pulls the cells back to the stress-free configuratiion, representing a state of cell-cell tension (Figure 2C). If the cells move further away from each other such that the outer ellipses no long overlap (i.e., $\delta <\delta_{\text{out}}$ ), then the cells are out of range from each other and stop transmitting force (Figure 2D); this state represents a release and lack of junctional connection between the 2 cells. These transmitted forces contribute two important aspects of collective migration to the model, with the extrusive force maintaining proper cell-cell spacing and the cohesive force maintaining the collective (and hence preserving the fluid barrier function of the endothelium). The amounts of extrusion and cohesion within the system are controlled with two energetic parameters: $W_{\text{push}}^{*}$ which defines the amount of work required push two adjacent cells together (i.e., δ→1 ), and $W_{\text{pull}}^{*}$ which defines the amount of work required to pull two adjacent cells apart (i.e., δ→−∞ ).

**FIGURE 3:**
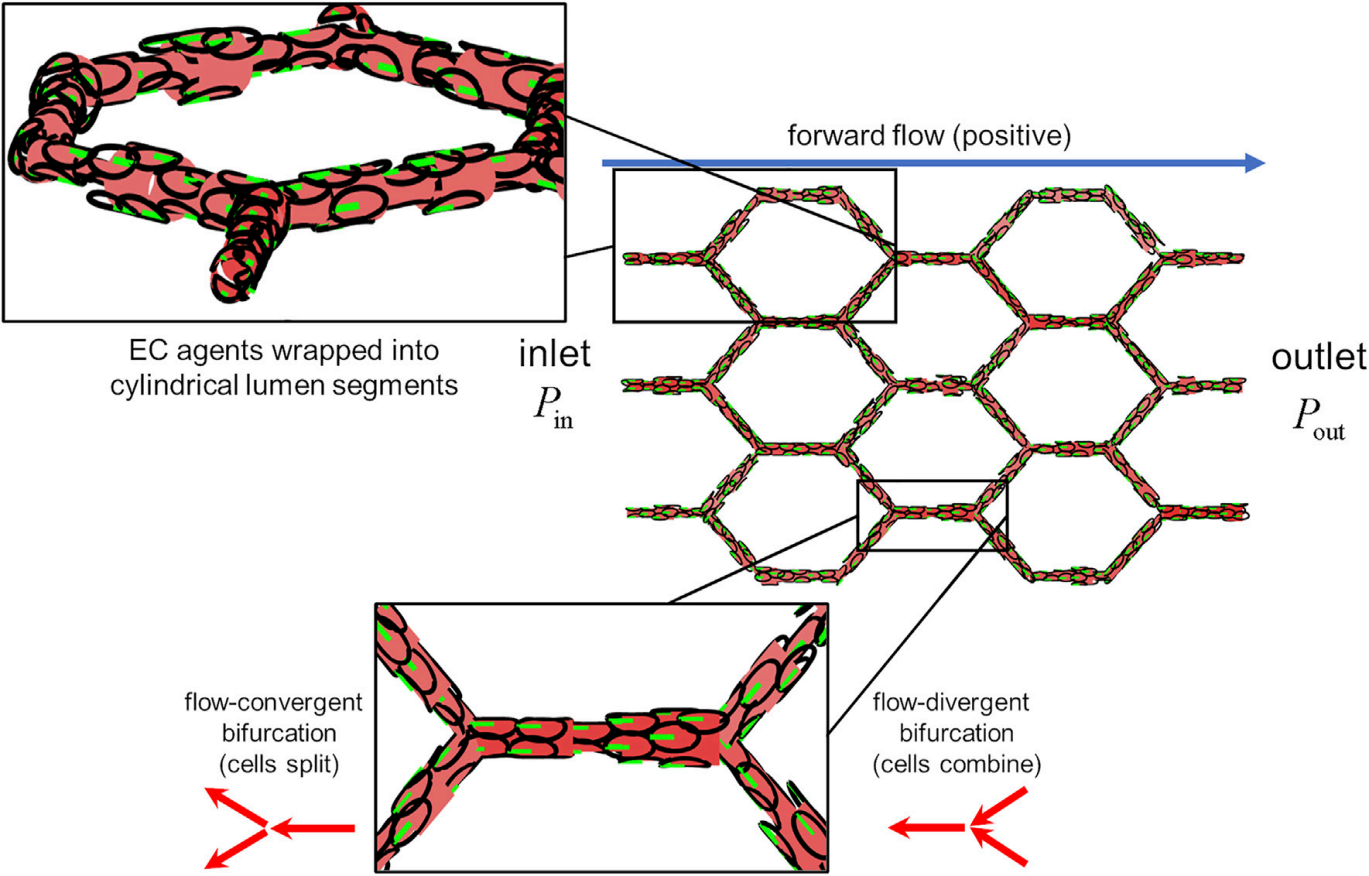
The idealised capillary plexus domain. Endothelial agents were confined to the surfaces of lumen segments, the diameter of which was determined by the number of occupant cells (upper inset). Pressure was prescribed at the inlet and outlet as to create forward flow (postive) along the +*x* direction. Cells align their polarity and migrate against the direction of flow (lower inset) At flow-covergent bifurcations, cells in the parent branch split evenly amongst the two child branches. At flow-divergent bifurcations, cells in the two child branches combine at the parent branch.

With our model, we present a novel coupling between blood flow and vascular structure *via* EC migration dynamics. Simulations were performed within an idealised capillary plexus domain with vessel edges of equal length arranged into a “honeycomb” pattern with characteristic length α (Figure 3; Supplementary Figure S3). Each vessel edge was discretised into cylindrical lumen segments, each the length of a single cell along its major axis. EC agents were confined to the surfaces of these cylinders, and the diameter of each lumen segment was a function of the number of cells currently occupying it: if the number of cells within a segment increases then the lumen diameter increases, and vice versa (Figure 3, upper inset). Pressure is prescribed at the inlet and outlet of the plexus to create forward flow approximated by the Hagen-Poiseuille equation, the magnitude and direction of which is determined by lumen diameter and the arrangement of cells within the plexus. ECs align their polarity against flow, and if at any point the flow reverses direction agents will similarly reverse their polarity. Cells apply force along their polarity in order to migrate, which we calculate using an overdamped model of cellular dynamics (with damping coefficient η ). In this migration model, the motion of each cell during a migration step is determined by the balance of forces acting on the cell (i.e., the sum of the migration force and all extrusive/cohesive forces from neighbours). In each simulation, all cells applied the same amount of migration force set by the parameter $k_{\text{mig}}$ producing a mean migration velocity amongst cells, $\bar{v}$ . ECs migrate upstream until the encounter a vessel bifurcation, where they split apart or combine depending on the state of flow (Figure 3, lower inset). At flow-divergent bifurcations, ECs simply combine at the parent vessel; at flow-convergent bifurcations, ECs are evenly split amongst the two child branches. Periodic boundary conditions were prescribed on migration such that cells that exit at the inlet re-enter at the outlet. We update cell positions during migration using a forward Euler’s scheme, in which we assume constant velocity for each cell over a discrete step in time ( Δt ). After each of these “migration steps,” we run an intercalation routine which allows cells to passively rearrange to minimise stress within the tissue (as our migration model assumes quasi-static equilibrium). After each migration and intercalation step, flow is re-calculated based on the updated cell positions and the model progresses to the next time step until the end of the simulation. An overview of the model can be found in Algorithm 1. We ran our model with 50 different random seed numbers (randomising the initial position of agents each time) at various values of extrusion $W_{\text{push}}^{*}=\left\{1/3,1,3,5\right\}$ and cohesion $W_{\text{pull}}^{*}=\left\{0,1,2,3\right\}/72$ , and characterised the changes in EC migration dynamics and network morphology and functionality associated with each set of parameters.

Algorithm 1 Agent-based model of flow-mediated collective migration driven by force transmission.

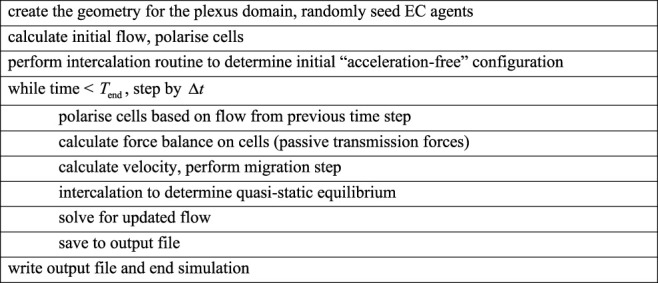

### Uniform Flow Polarity–Extrusion Stabilises Whilst Cohesion Disrupts

The first question we aim to answer in our study is: 1) what changes in force transmission between migrating ECs cause cells to aggregate and distribute unevenly along a minority of flow pathways as seen in AVMs? To answer our question, we will vary force transmission parameters and assess the impact on individual EC migration dynamics and the vascular plexus as a whole. In order to simplify our analysis, we will initially consider a uniformly polarised population of cells (against flow).

**FIGURE 4:**
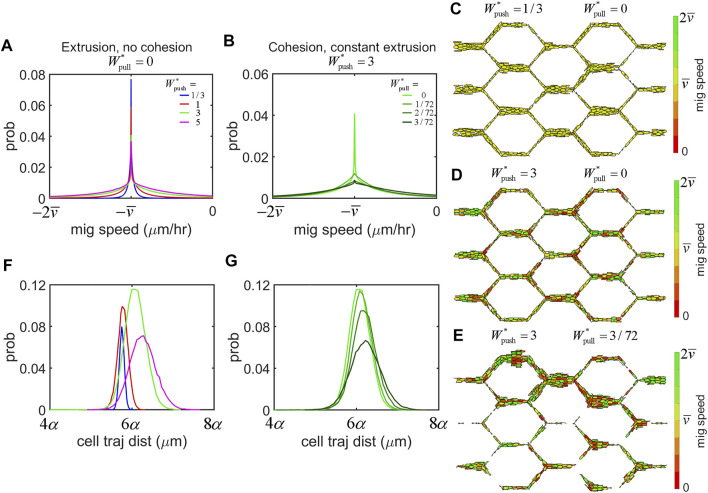
Force transmission regulates EC migration dynamics. **(A)** Histrogram of migration speeds amongst the cell population across all simulations were centred at the mean migration speed, $\bar{v}$ . In simulations without cohesion ( $W_{\text{pull}}^{*}=0$ ), increasing extrusion increased the spread of migration speeds amongst cells. **(B)** Similarly, in simulations with constant extrustion ( $W_{\text{push}}^{*}=3$ ) increased cohesion flattened the distribution of migration speeds amongst cells further **(C)** Simulations with low extrusion (i.e., $W_{\text{push}}^{*}<1$ ). resulted in near uniform migration speed of $\bar{v}$ amongst ECs throughout the plexus (yellow). **(D)** Increased extrusion resulted in more cells moving at speeds faster (green) and slower (red) than $\bar{v}$ . **(E)** Adding cohesion further increased heterogenity in migration speeds amongst cells throughout the plexus. **(F)** The cell trajectory distance measures the total distance travelled by cells during the simulation. Interestingly, in simulations without cohesion cells travelled further with increased extrusion as seen by a shift to the right in the distributions of cell trajectory distance. **(G)** Similarly, increased cohesion caused ECs to travel further as seen in similar shifts to the right in the distributions of cell trajectory distance.

We found that EC migration dynamics were highly sensitive to force transmission between cells, even with each cell applying identical amounts of migratory force. In simulations without cohesive force (i.e., $W_{\text{pull}}^{*}=0$ ), the distribution of migration speeds amongst cells was centred at $-\bar{v}$ (with the negative sign implying migration against the direction of flow) (Figure 4A). Increasing extrusive forces amongst cells increased the spread of the distribution of migration speeds amongst cells, with more cells moving both faster and slower than $\bar{v}$ . Adding cohesive forces to the system flattened out the distribution of cell migration speeds even further (Figure 4B). A cell migrating at speeds different from $\bar{v}$ indicates interference from forces transmitted by its neighbours. At values of $W_{\text{push}}^{*}<1$ , migration forces dominate over extrusion forces and the majority of cells migrate without interferece at a speed of $\bar{v}$ (Figure 4C). However, at values of $W_{\text{push}}^{*}>1$ extrusion forces are stronger than migration forces and we find heterogenous distribution of migration speeds amongst the cell population (Figure 4D). Note that this interference can cause cells to migrate either faster than or slower than $\bar{v}$ . Increased cohesive forces created further heterogeneity within the cell population (Figure 4E). Lastly, we can quantify the overall effectiveness of cell migration by measuring the total distance cells travelled during the simulation (defined as the cell trajectory distance). Surprisingly, we found that increased amount of pushing between cells allowed the cells to travel further during migration, as seen in a shift to the right in distributions of cell trajectory distance as we increased $W_{\text{push}}^{*}$ (Figure 4F). We found a similar shift to the right (albeit less so) and flattening of distributions of cell trajectory distances with increased amounts of cohesion as well (Figure 4G).

**FIGURE 5:**
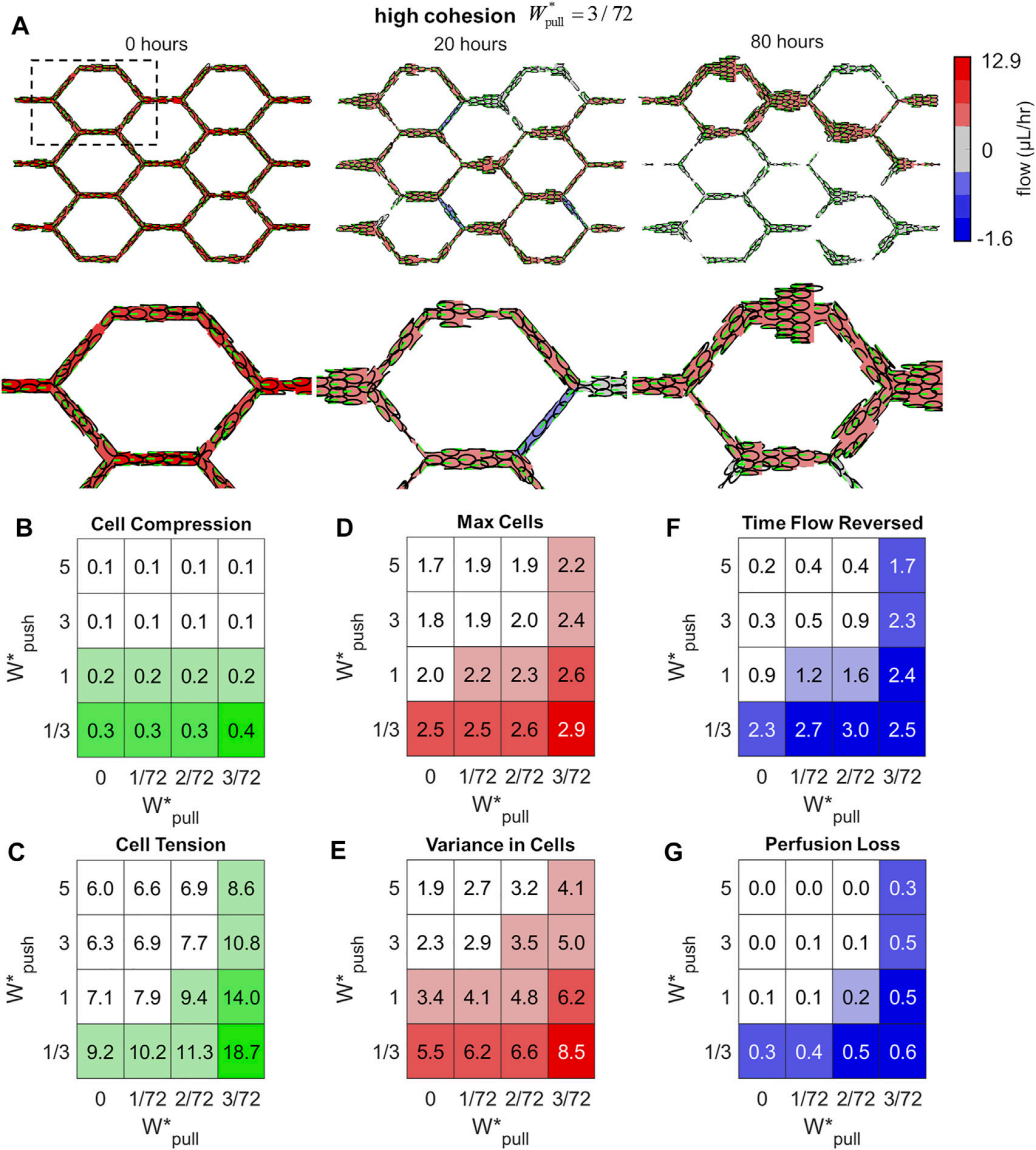
Excess cohesion promotes shunt formation. **(A)** Snapshots from an example simulation at high cohesion ( $W_{\text{push}}^{*}=3$ ; $W_{\text{pull}}^{*}=3/72$ ). Forward flow is presented in red, reversed flow in blue, and no flow in grey. An example of a simulation at high cohesion is presented here, with the whole plexus domain in the top row and zoomed in region of a single honeycomb cell on the bottom row (zoomed in region indicated by dashed line in top left panel). In simulations with high cohesion, cell distribution tended to become skewed as cells aggregated along a minority of flow pathways, leading to a loss of perfusion and functional shunting **(B–G)** Mean values of features at each value of $W_{\text{push}}^{*}$ and $W_{\text{pull}}^{*}$ ; values above the median are coloured while values below the median are colourless. Features associated with shunt formation decreased with extrusion (moving up columns) and increased with cohesion (moving across rows). Extrusion tended to reduced stress within the endothelium while cohesion prevented stress dissipation, as seen in values of Cell Compression **(B)** and Cell Tension **(C)**. Cohesion also tended to aggregate cells and skew the distribution throughout the plexus, as seen as increased Max Cells **(D)** and Variance in Cells **(E)** amongst vessels; extrusion tended to stabilise the plexus and ensure that cells remained evenly dsitrubted. Simulations with high cohesion experienced more prolonged flow reversal events, as the Time vessels spent with Flow Reversed increased **(F)**, whilst flow reversals in simulations with high extrusion were short-lived. Lastly, the percentage of flow pathways which lost flow during remodelling increased greatly with cohesion, indicated by pronounced levels of Perfusion Loss **(G)**; perfusion was perserved in simulations with higher levels of extrusion.

Our primary interest is to assess how force transmission between individual ECs influences the remodelling outcome as it emerges within the vascular network as a whole. In our study, we found that we could push our system from stable perfusion to shunt formation *via* control of a single parameter: the amount of cohesion within the system, $W_{\text{pull}}^{*}$ . Cells remained evenly distributed throughout the plexus in simulations without cohesion and perfusion remained largely interrupted (Supplementary Figure S4; Supplementary Video S1). However, in simulations at high cohesion cells tended to aggregate along a small percentage of flow pathways, depleting both cells and flow from other regions of the plexus (i.e., functional shunting) (Figure 5A; Supplementary Video S2).

We defined six network-level features in order to characterise the state of the vascular network and the emergence of functional shunting during remodelling. We measured the mean Cell Compression (CC) and mean Cell Tension (CT) amongst the cell population (indicated by positive and negative overlap between agents) to assess levels of stress carried within the endothelium tissue. The idealised capillary plexus initially has cells evenly distributed throughout the plexus; any loss of this even distribution will be indicated by measurements of the Max number of Cells amongst vessels (MC) which increases if any particular vessels is growing larger than average, and the Variance in Cell number amongst vessels (VC) which increases if the distribution of cells within the plexus becomes uneven. Lastly, we calculated two features to indicate flow disruption during remodelling: changes in flow direction are indicated by the time vessels spent in Flow Reversed (FR), and Perfusion Loss was calculated as the percentage of flow pathways in which flow dropped to zero (PL). Definitions of each of these features can be found in the Methods section. In general, we found that all of our features were strongly associated with shunt formation and tended to decrease as we added extrusion to the system and increase as we added cohesion. When cells generated strong extrusive forces, they were able to effectively intercalate to reduce stress within the tissue more effectively as seen in decreased levels of Cell Compression (Figure 5B) and Cell Tension (Figure 5C); increased cohesive forces interferred with this intercalation and produced greater levels of tension between cells.

Extrusion and cohesion dramatically impacted the distribution of cells throughout the plexus during remodelling as well. Increased levels of extrusion prevented vessels from growing too large, as seen in decreased values of Max Cells (Figure 5D), and ensured that cells remained evenly distributed throughout the plexus, as seen in reduced Variance in Cells (Figure 5E). Conversely, increased cohesion produced large aggregates of cells and skewed the distribution within the plexus, as seen in increased values of Max Cells and Variance in Cells amongst vessels. Excess cohesion tended to disrupt flow and perfusion during remodelling whilst extrusion stabilised. Vessels in simulations with high cohesion experienced more prolonged episodes of flow disruption, as seen in increased Time vessel spent with Flow Reversed (Figure 5F); simulations with high extrusion experienced some flow reversal and loss but these events were short-lived in comparison. Lastly, the percentage of flow pathways which lost flow during remodelling dramatically increased with cohesion, as seen in increased Perfusion Loss (Figure 5G), whilst perfusion was stabilised with increased extrusion.

We used Pearson’s correlation coefficient to assess the overall relationship between our network-level features of shunt formation with various parameters within the model. In general, features of shunt formation were negatively correlated with $W_{\text{push}}^{*}$ , demonstrating the overall stabilising effect of extrusion (Supplementary Figure S5A). This correlation weakened at higher values of $W_{\text{pull}}^{*}$ , suggesting that extrusion had a reduced effect in the face of high cohesion. Conversely, our features were positively correlated with changes in $W_{\text{pull}}^{*}$ demonstrating the general disruptive effect of cohesion (Supplementary Figure S5B). Correlation with $W_{\text{pull}}^{*}$ was weaker at lower values of $W_{\text{push}}^{*}$ as cohesive forces only kick in at negative overlap, and ECs with low $W_{\text{push}}^{*}$ exist in a state of mostly positive overlap. The cohesive parameter $W_{\text{pull}}^{*}$ is itself a combination to the linear force transmission parameter $k_{\text{coh}}$ and nonlinear yield parameter Γ . Shunt formation was postively correlated with both $k_{\text{coh}}$ while holding Γ constant (Supplementary Figure S5B) and Γ while holding $k_{\text{coh}}$ constant (Supplementary Figure S5C). However, we found that varying these parameters together whilst keeping $W_{\text{pull}}^{*}$ constant produced no discernable impact on shunt formation (Supplementary Figure S5D). These results suggest that within the range of these two parameters that we tested, it is the energetic combination of these two parameters ( $W_{\text{pull}}^{*}$ ) that exerts control on shunt formation. Intra-feature correlation analysis also revealed that the majority of our shunt features correleated well with each other, with the exception of Cell Compression which seems to play a limited role in shunt formation (Supplementary Figure S5E). All of our features correlated strongly with perfusion loss (PL), demonstrating their effectiveness as indicators of functional shunting. We found a strong association between cell distribution and cell tension (CT and MC, VC), indicating that cells aggregating into shunts were under increased amounts of tension. Lastly, we found a strong association between flow reversals and perfusion loss (FR and PL), suggesting that flow reversals during remodelling may be playing a mechanistic role in shunt formation and perfusion loss. Lastly, all of our findings on the regulation of EC migration dynamics and network stability were consistent within uniformly polarised populations of cells, whether the cells were polarised against flow or with flow the results were similar (data not shown).

There were several additional parameters which had no impact on shunt formation within our model. The total amount of iterations spent in the intercalation scheme (needed to resolve quasi-static equalibirum amongst the cells after each migration step) tended to increase with both extrusion and cohesion; however, the Total Intercalation Iterations had no impact on shunt formation (Supplementary Figure S6). Lastly, changes in the damping coefficient in the dynamic model, η , and the Euler time step size, Δt , both produced no effect on shunt formation (Supplementary Figures S7, 8).

### Flow Reversals as a Mechanism of Shunt Formation

**FIGURE 6:**
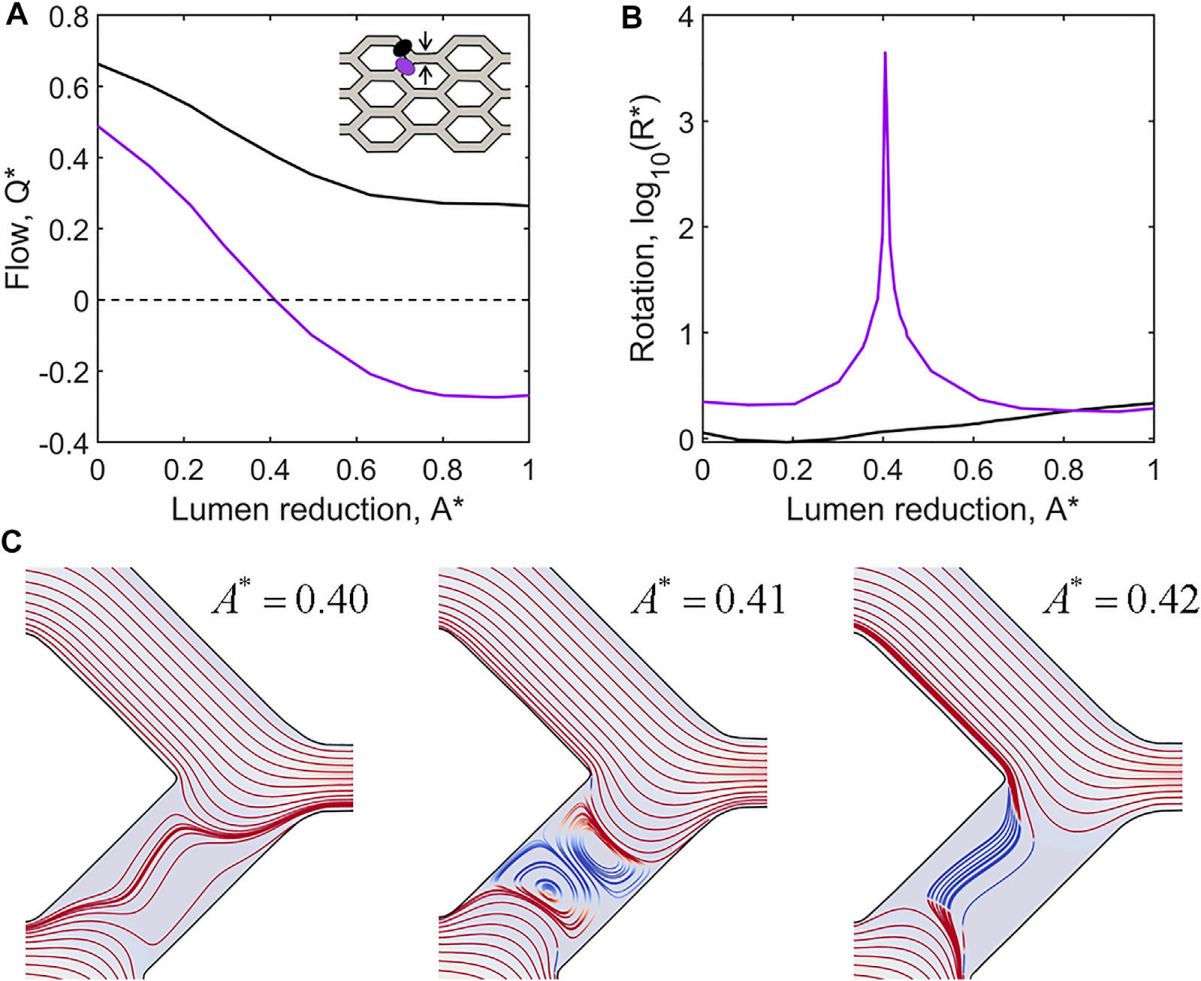
Flow reversals and rotation occur in adjacent vessels as a result of lumen reduction. **(A)** CFD simulations of flow within the idealised capillary plexus subject to a full collapse of the upper middle vessel pathway (as indicated by black arrows in the inset diagram). Normalised flow $Q^{*}$ in the upper feeding branch is shown in black, while $Q^{*}$ for the lower feeding is shown in purple. The state of vessel collapse is given as lumen reduction, $A^{*}$ , which represents the percentage by which the vessel lumen has been reduced. The flow in both feeding branches reduces as lumen reduction increases. Of special note is that the flow in the lower feeding branch (purple) reaches zero at $A^{*}=0.41$ and becomes increasingly negative upon further lumen reduction, indicated flow reversal in this branch. **(B)** We also found a spike in normalised fluid rotation, $R^{*}$ , occurring at $A^{*}=0.41$ upon which flow begins to switch from forward (positive) to reversed (negative). **(C)** Velocity stream lines displaying forward flow in red and reversed flow in blue demonstrate the progression from forward flow ( $Q^{*}>0$ ) at $A^{*}=0.40$ , recirculation ( $Q^{*}=0$ ) at $A^{*}=0.41$ , to reversed flow ( $Q^{*}<0$ ) for $A^{*}>0.41$ .

One of the most striking observations in simulations of shunt formation was the presence of prolonged flow reversals within remodelling plexuses. Flow reversals occur as changes in vessel diameter modulate the paths of least resistance. Analysis of our honeycomb plexus model *via* computational fluid dynamics indicate that reduction of lumen area greater than 40% relative to neighbours was sufficient to create flow reversals (Figure 6A). Additionally, we observed pronounced fluid rotation and the formation of lid driven cavity vortices during flow reversals, suggesting significant flow disruption and potentially complex shear signalling to luminal ECs (Figures 6B,C). Notably, we found flow reversals only occurred in conjunction with vessel contraction; vessel expansion did not impose flow reversals, indicating that luminal collapse is the key driver of flow reversals within the plexus during remodelling.

**FIGURE 7:**
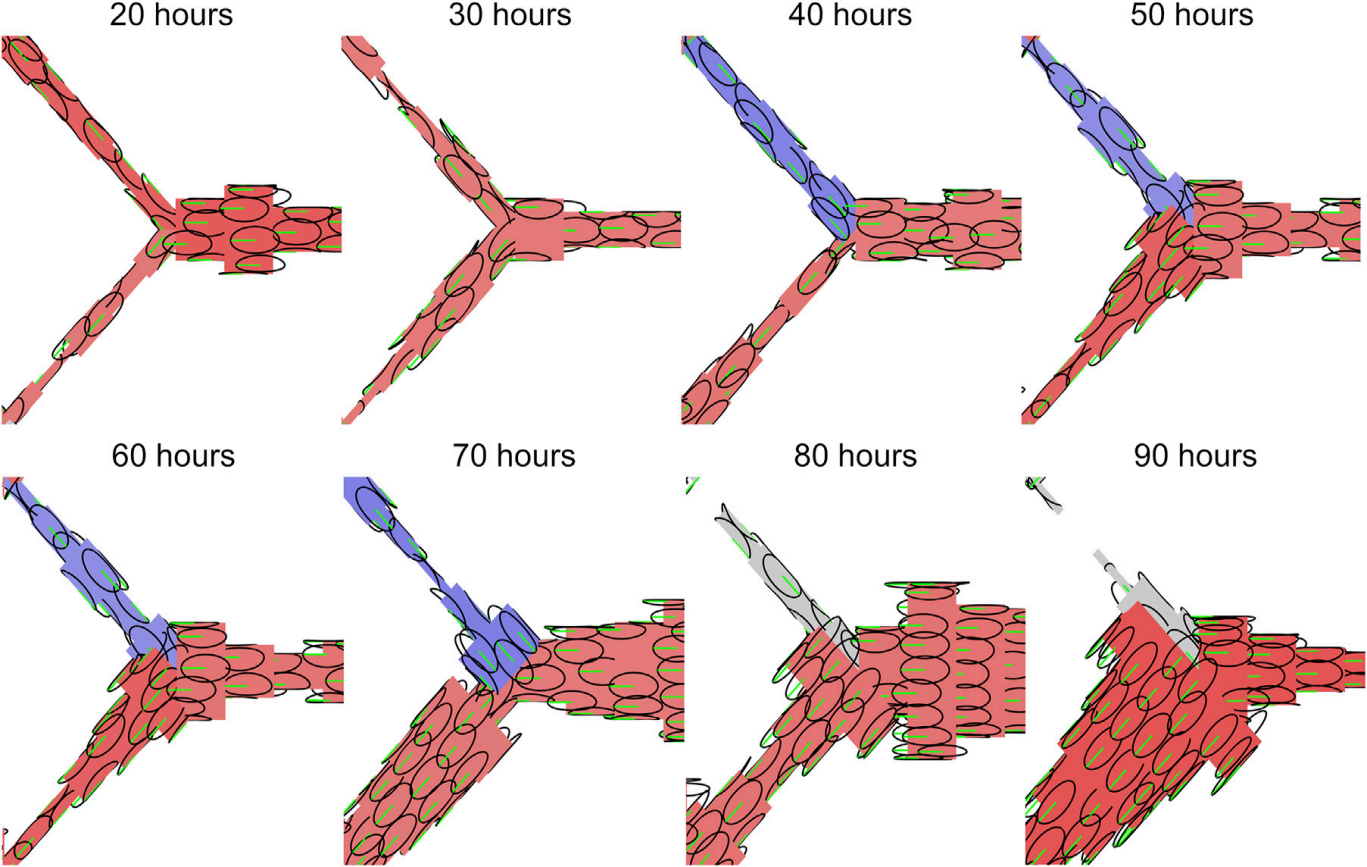
Flow reversals switch EC migration paths at vessel bifurcations. Inner ellipse of individual EC agents shown in black, polarisation and migration direction shown in green. Vessels with forward flow are displayed in red, reversed flow in blue, and no flow in grey. Flow reversals switch flow-convergent bifurcations, where EC migration paths split, to flow-divergent bifurcations where EC migration paths combine. Prolonged flow reversals gives ECs enough time to aggregate along a minority of flow paths, depleting both cells and flow from other regions of the plexus resulting the functional shunting.

Flow-mediated remodelling is driven by ECs migrating against the direction of flow. Thus, flow reversals can exert powerful control over the remodelling outcome as they impose changes on migration direction. Most importantly, flow reversals switch flow-convergent bifurcations, where EC migration paths diverge, to flow-divergent bifurcations where EC migration paths combine. Flow reversal events occurred in all our simulations as EC migration reduced lumen diameter at various locations within the plexus. Extrusive forces allow ECs to intercalate and restore lumen diameter, resolving flow reversals quickly. However, high levels of cohesion prevent ECs from intercalating as effectively and allow flow reversals to persist long enough to have drastic consequences on migration patterns at bifurcations (Figure 7). Prolonged flow reversals cause splitting ECs to instead combine, and in conjunction with high cohesion results in ECs clustering together into large aggregates along a minority of flow pathways, depleting ECs and thus perfusion from the rest of the plexus (i.e., functional shunting).

### Mixed Flow Polarity–Extrusion Creates Traffic Jams

**FIGURE 8:**
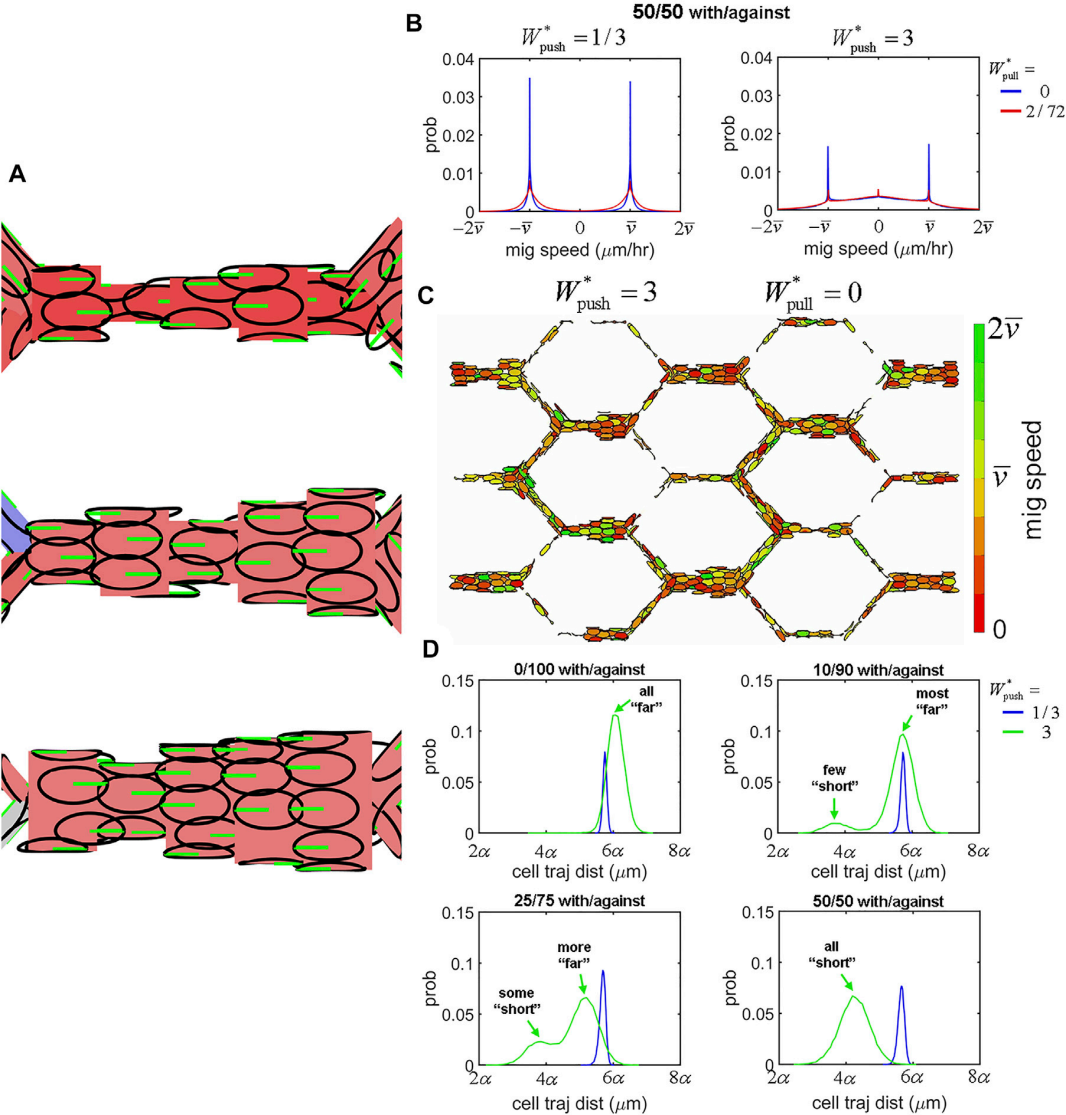
EC migration dynamics within mixed polarity populations. **(A)** Seeding simulations with a mixture of cells polarised either with or against flow led to difficulty for cells with opposing polarity to move past each other during migration. **(B)** Opposing polarisation created interference in migration at increased levels of extrusion. At low extrusion ( $W_{\text{push}}^{*}=1/3$ ), opposing cells could easily move past each other and interfered with each other very little (left). At a mixture ratio of 50/50 with/against flow, this resulted in migration speeds even distributed at peaks of $-\bar{v}$ (against flow) and $\bar{v}$ (with flow). Adding cohesion to the system flattened the distributions slightly but did not change the overall nature of migration speed amongst cells. Migration speeds at high extrusion ( $W_{\text{push}}^{*}=3$ ) had similar peaks at $-\bar{v}$ and $\bar{v}$ , but also many more cells migrating at speeds in between (right). This indicates slower migration amongst cells and increased interference amongst opposing cells. Adding cohesion created further traffic jams amongst mixed polarity populations of cells. **(C)** High extrusion within mixed populations led to depletion of cells at some locations in the plexus due to traffic jams and cells being unable to move past each other effectively. **(D)** Cells migrating in mixed polarity populations tended to separate into classes of “far” migrators and “short” migrators as indicated by high and low values of cell trajectory distance. All cells were “far” migrators at low extrusion as they were able to move past each other effectively (blue). As the ratio of opposing migrators increased, the percentage of cells that were “short” migrators increased until at a 50/50 with/against flow mix all cells were “short” migrators.

One of the most profound observations upon Alk1 deletion in the postnatal mouse retina model was a switch in capillary cells from polarisation against the direction of blood flow to a mixture of polarisation with/against flow. Therefore, the second question we set out to answer in our study was: 2) what are the effects of introducing mixtures of cell polarity on EC collective dynamics and angiogenic remodelling? We created four different mixed populations of cells by randomly assigning cells to either migrate “with flow” or “against flow” at the following ratios: 0/100 (i.e., uniformly polarised against flow), 10/90, 25/75, and 50/50. We found that inserting opposingly polarised cells into the collective dramatically impacted migration dynamics and vascular morphology, even at relatively small percentages. Opposingly polarised cells tended to create “traffic jams” during migration as cells had difficulty moving past each other (Figure 8A). This difficulty seemed to increase with extrusion. While extrusion was a stabilising force during migration of uniformly polarised cells by allowing ECs to intercalate more effectively and resolve vascular malformations, in the presence of opposing polarisation extrusion had the oppose effect: instead of stabilising, extrusion tended to destabilise. At low levels of extrusion ( $W_{\text{push}}^{*}<1$ ), cells were able to move past each other effectively and cells migrated at speeds near $\pm \bar{v}$ (Figure 8B; Supplementary Video S3). However, at higher levels of extrusion more cells tended to migrate at speeds closer to 0, indicating a significant slowdown of migration. Adding cohesion to the system in these cases seemed to enhance the traffic jamming. These traffic jams resulted in cells getting stuck in aggregates, depleting other portions of the plexus of cells leading to vascular malformation and instability (Figure 8C; Supplementary Video S4). Mixed polarity populations of cells seemed to separate into “far” migrators (cells that travelled long distances during migration as seen by a higher cell trajectory distance) and “short” migrators (cells that travelled a much shorter distance during the migration). At low levels of extrusion, all cells were “far” migrators as there was little interference due to opposing polarisation (Figure 8D, blue). However, at high levels of extrusion two classes of cell migrators began to separate: at 0/100 with/against all cells were “far” migrators. At mixtures of 10/90 and 25/75 with/against, small populations of “short” migrators began to emerge (Figure 8D, green). Lastly, at 50/50 with/against all cells were “short” migrators as no cells were able to escape interference from opposing migrators. Plotting the clusters of cells migrating “with” and “against” flow revealed that it was the minority of “with” cells that became “short” migrators as they attempted to penetrate the larger majority of opposingly polarised neighbours (Supplementary Figure S8).

It would seem that the role of extrusive forces is completely changed within mixed polarity populations of cells. Extrusion, whilst normally decreasing and stabilising most of our network-level features of shunt formation, now instead increased them dramatically: Cell Tension, Max Cells, Variance in Cells, and Perfusion Loss all increased significantly with extrusion within mixed polarity populations (Supplementary Figure S9). While extrusion was negatively correlated with shunt formation in uniform polarity populations, in mixed polarity populations extrusion was instead positively correlated with shunt formation, with increasing amounts of positive correlation as the ratio of opposingly polarised cells increased (Supplementary Figure S10 top row). Cohesion remained positively correlated with shunt formation within mixed polarity populations, although it is role seems to be dramatically reduced in these cases (Supplementary Figure S10 bottom row). Lastly, we found a strong correlation between all our features in our simulations within uniform polarity populations, with special note of a strong correlation between Flow Reversals and Perfusion Loss indicating a mechanistic role for changes in flow direction in functional shunting. However, in the presence of mixed polarity populations this correlation between Flow Reversals and Perfusion Loss vanished by increasing amounts as the ratio of opposingly polarised cells increased (Supplementary Figure S11). These last findings suggest that flow reversals are not the primary mechanism for vascular malformation and instability in mixed polarity populations; instead, we believe extrusive forces in the presence of opposingly polarised cells to be the cause in these cases. In the altered case of mixed polarity amongst the cell population, such as the case found in Alk1 deletion in the mouse retina model, the once stabilising presence of cell extrusion is turned on its head and instead becomes an adversary to vascular stability and function.

## Discussion

With our novel computational approach, we have demonstrated the role of force transmission in the cellular mechanics driving collective migration during angiogenic remodelling and shunt formation. The parameter $W_{\text{push}}^{*}$ describes the energy required to push cells together and scales the resistance (i.e., stiffness) to cell-cell compression *via* extrusive forces. We found that extrusive forces within a uniformly polarised population stabilised the vasculature, allowing cells to intercalate with ease to reduce stress and resolve diameter imbalances, preserving perfusion and maintaining symmetry and an even distribution of cells throughout the plexus. The parameter $W_{\text{pull}}^{*}$ describes the energy required to pull cells apart and scales the strength of cell-cell adhesion (i.e., stickiness or friction experienced by ECs as they attempt to move past one another) *via* cohesive forces. We found that high levels of cohesive force between cells during collective migration disrupted the vasculature by interfering with EC intercalation. This interference created tension within the endothelium tissue, prevented diameter imbalances from being resolved which allowed short-lived stochastic flow reversals (appearing due to network effects arising from these imbalances) to persist, causing cells to cluster and aggregate *via* a change of traffic patterns at vessel bifurcations. Aggregation due to high cohesion robbed the remaining regions of the plexus of both cells and perfusion, resulting in functional shunting in which the majority of flow accumulated along a minority of flow pathways.

All of these aspects of shunt formation were captured in aggregate behaviour of our six network-level features during parameter sensitivity analysis. In general we found that high cohesion tended to have a more disruptive effect at lower levels of extrusion, indicating that extrusive forces can work to balance out the effects of high cohesion to a certain degree. However, correlation analysis revealed a weakened role of extrusion in the face of high cohesion, indicating that extrusive forces can only do so much to counteract these effects. Interestingly, we found the highest values of our shunt features in simulations at low extrusion ( $W_{\text{push}}^{*}$ = 1/3) in which ECs are less able to resist compression and enforce proper spacing during intercalation. These findings indicate that the mechanisms which ensure proper spacing between ECs during collective migration play an important role in the effects we found. Whether due to a lack of extrusion or an excess of cohesion, the inability of ECs to achieve proper spacing: 1) increased stress within the endothelial tissue, 2) skewed the distribution of cells around vessel lumens which results in uneven splitting of cells at bifurcations, 3) prolonged flow reversals which combined EC migration paths instead of split, and 4) created aggregates of cells which produced functional shunting.

Overexpression of integrins β1, α5, and αv is a feature within AVMs in Alk1 deficient mice (Park et al., 2021). In fact, inhibition of integrins αvβ3, αvβ5, and α5β1 upon induced knockdown of Alk1 reduced AVM formation (Park et al., 2021), indicating that integrin activity plays a role in AVM formation. In our model, we produced functional shunts resembling AVMs with either excess cohesion between ECs or mixtures of cell polarity. At the centre of both these mechanisms are cell-cell adhesions, which are involved in flow sensing/polarity and must remodel dynamically to allow rearrangements of cells during vascular patterning (that is, ECs must hold each other but not too tightly (Szymborska and Gerhardt, 2018)). At first, it might not be immediately clear how the experimental observation of integrin overexpression in AVMs connect with our demonstrated role of cell-cell adhesions. However, there is an ever-increasing collection of evidence suggesting a strong linkage between cell-cell adhesion, integrin binding, and polarity in endothelial flow-migration coupling. Junctional complexes are involved in shear stress mechanosensing (Szymborska and Gerhardt, 2018), and EC polarity and orientation are regulated by VEGF signalling which also plays a role in adherens junction remodelling in the presence of shear stress (Vion et al., 2021). Nuclear-Golgi polarity is also dependent on integrin binding to ECM proteins and Cdc42 signalling (Etienne-Manneville and Hall, 2001; Tzima et al., 2003; Zovein et al., 2010). Vinculin is an important regulator of integrin-adherens crosstalk which also controls junction dynamics and EC collective behaviour, and disruption of which induces vascular malformations during angiogenesis (Mui et al., 2016; Carvalho et al., 2019; Kotini et al., 2021). Integrins β1, β3, and β5 are required for of EC barrier function (Su et al., 2012; Pulous and Petrich, 2019), which is regulated by adherens junctions between cells. Absence of αvβ3, αvβ5, and α5β1 all resulted in a loss of barrier function and integrity of ECs (Pulous and Petrich, 2019). Additionally, integrins αvβ3 and α5β1 have been found localised at cell-cell contacts (Lampugnani et al., 1991; Su et al., 2012). If an absence of these integrins results in a loss of EC cohesion, than overexpression of these integrins in Alk1 mutant ECs could result in excessive EC cohesion. Furthermore, integrins are potent regulators of YAP/TAZ (Dupont, 2016), which in turn regulate the morphology and turnover of adherens junctions (Neto et al., 2018). Alk1 deletion in ECs results in increased expression and nuclear localisation of YAP/TAZ, with high expression of YAP/TAZ as well as integrins β1, α5, and αv within AVMs (Park et al., 2021). Together, this evidence along with our modelling results suggests that induced deletion of Alk1 signalling in mutant mice results in dysfunctional cohesion between ECs in an integrin- and YAP/TAZ-dependent manner which can result in AVMs similar to the functional shunts produced in our computational model at high values of $W_{\text{pull}}^{*}$ .

The complement of EC cohesion are extrusive forces, scaled in our model by the parameter $W_{\text{push}}^{*}$ . Extrusion controls the resistance of ECs to compression and the amount of “pushing” cells exert on each other as they attempt to occupy the same space. In the *Drosophila* ovary, collectively migrating epithelial cells activate cycles of myosin II contraction to resist compression and nagivate through physical restrictions (Aranjuez et al., 2016). This role of myosin II in resisting compression from neighbouring cells is akin to the extrusion forces within our model. Extrusion forces within uniformly polarised populations were a stablising force, improving EC migration and promoting intercalation to reduce stress, resolve diameter imbalances, and perserving vascular perfusion. However, a common trait described amongst Alk1 deficient ECs in AVMs is a loss of flow-based polarity (which itself may arise from dysfunction between cell-cell adhesion, integrin binding, and migration direction as described previously), with cells found with a mixture of polarities both with and against flow (Rochon et al., 2016, 1; Park et al., 2021). We found that introducing mixtures of cell polarity into our model produced dramatic effects on force transmission and collective migration, even at relatively small percentages of opposingly polarised cells. The presence of mixed polarity switched the role of cell-cell resistance to compression from a stabilising factor to destabilising. High levels of extrusion within mixed polarity created traffic jams, preventing effective migration by decreasing migration speeds towards zero and reducing the distance ECs were able to travel overall. Areas of these traffic jams may act as nucleation sites for AVMs as they promote aggregation of ECs similar to what we found in simulations with high levels of cohesion. The take-away message from our findings are that disruption of normal force transmission between ECs, which we produced *via* high levels of cohesion or by inserting mixtures of cell polarity, resulted in vascular malformation and functional shunting during angiogenic remodelling. Our findings also suggest that cell-cell adherens junctions, which play a governing role in both mechanisms, may be a useful target in the treatment and prevention of vascular disease and AVMs in the future.

Our mathematical methodology provides a novel and useful new platform for the study of endothelial tissue mechanics in flow-mediated collective migration within a multiscale perspective. In these methods, we prescribe behaviour at the cellular microscale (i.e., parameters regulating force transmission between individual ECs) and observe the emergent remodelling outcome at the tissue macroscale (i.e., the vascular plexus as a whole). Although our model of cellular mechanics *via* overlapping ellipses is relatively simplistic, it is capable of producing complex biomechanical constitutive behaviour at the level of the tissue continuum. The tissue constitutive model can be considered as a spatially heterogeneous material with strain energy derived from overlap between EC agents. This material exhibits tension-compression nonlinearity *via* the moduli $k_{\text{coh}}$ and $k_{\text{ext}}$ . Migration dynamics combined with the yield stretch parameter Γ adds an element of plasticity to material as well, as “yielding” of junctional connections between migrating cells produces entropic rearrangements and history effects. Our model also includes fluid-structure interactions, uniquely coupled through network flow and EC migration dynamics. All of these behaviours would be quite difficult to describe constitutively from the “top-down” at the tissue continuum level, yet emerge quite naturally from the “bottom-up” through our simple agent-based cellular mechanics model.

However, the work we present here still relies on simplifying assumptions necessary to make the parameter space tractable, and there are many important traits of the physical system that have yet to be considered but may prove useful additions in the future. We modelled blood as a Newtonian fluid and Poiseuille flow, but in reality blood exhibits many important attributes of non-Newtonian fluids. Specifically, the inclusion of erythrocytes within our network haemodynamics may be crucial next step, as we have previously identified an important association between erythrocyte dynamics and vascular remodelling due to plasma skimming effects (Zhou et al., 2021). Our current model also included a simplistic representation of EC flow-migration coupling as well, with agents migration governed by $k_{\text{mig}}$ which we kept constant in this study. The concept of “set point theory” in vascular remodelling proposes that ECs seek an optimal amount of shear stress, and deviations away from this optimal level promote a change from quiescence to the remodelling phenotype (Baeyens and Schwartz, 2016). Further, there is evidence that EC migration speed follows a “band pass-like” behaviour as a function of shear stress in which EC migration slows down and stops all together for shear stress values that are too high or low flow (Tabibian et al., 2020). This complicated relationship between migration speeds and shear stress could result in the “locking in” of ECs within vessels with exceptionally high or low shear stress (the latter of which may be relevant inside of large diameter AVMs) and result in a gradient of migration speeds across the vascular plexus (from venous to arterial) during remodelling. This behaviour could be implemented in our model in future studies by describing the migration force parameter $k_{\text{mig}}$ as a function of stress. We chose an elliptical representation of ECs as Tabibian et al. demonstrated that EC elongation was a critical aspect of angiogenic remodelling (Tabibian et al., 2020). However, in this current study we kept the extend of elongation constant; in future studies it might be interesting to vary the extent of EC elongation as a function of shear stress and other singalling regulators. Further, we could move our repesentation of ECs away from idealised ellipses towards a more intricate representation of EC geometry *via* meshless methods and introduce shear stress and Notch/VEGF singalling as important regulators of intracellular mechanics and cell-cell adhesion (Bentley et al., 2014; Vion et al., 2021). The inclusion of integrin binding and ECM mechanics may be worth considering in the future as well. Finally, our model lacked an explicit representation of mural cells within the capillary plexus (i.e., vascular pericytes). Pericytes play an important role in protecting the integrity of the vascular barrier and promoting EC junction formation (Zhao and Chappell, 2019). Therefore, defective EC-pericyte interactions could be an important driver of force transmission and EC cohesion during angiogenic remodelling and AVM. Indeed, defective TFG-β/BMP signalling in ECs (which involves Alk1) has been shown to affect vessel stability and pericyte attachment (Thalgott et al., 2015), further emphasising a role for defective EC-pericyte interactions during the onset and progressing of AVM. Future expansion of our computational platform into a multi-agent model which includes pericyte agents would allow us to interrogate the dynamics of pericyte coverage during angiogenic remodelling and provide new insight into this emerging key player in HHT and AVM.

In summary, we have demonstrated the important role of force transmission at cell-cell adhesions regulating flow-mediated EC collective migration during angiogenic remodelling. Excessive cohesion between migrating ECs led to aggregation and functional shunting, caused by prolonged flow reversals which change traffic patterns at vessel bifurcations. Mixed polarity within the EC population changed the nature of force transmission, with extrusion forces which normally stabilised the vasculature because a destabilising force, preventing effective migration as opposingly polarised ECs met resistance when trying to migrate past one another. Our unique computational framework provides novel insight into the collective dynamics of ECs during angiogenic remodelling not available within the confines of current *in vivo* experimental techniques, and should provide a useful platform for future studies in uncovering the mechanisms which may be used to prevent and treat AVMs.

## Materials and Methods

### Producing AVMs in Mouse Retinas *Via* Induced Alk1 Deletion

We induced AVMs in mouse retinas of Mfsd2a CreERT2 mice (kindly provided by Dr Bin Zhou at University of Chinese Academy of Sciences) by induced deletion of Activin receptor-like kinase 1 (Alk1) using *Alk1* ^*f/f*^ mice (kindly provided by Dr Paul Oh at Barrow Neurological Institute). All animal experiments were performed under a protocol approved by Institutional Animal Care Use Committee of Yale University. Gene deletion was induced by intra-gastric injections with 100 μg tamoxifen (Tx, Sigma, T5648; 2.5 mg/ml) into pups at P4 (experiments hence referred to as Alk1 iKO). Tx-injected CreERT2 negative littermates were used as controls (experiments hence referred to as Controls).

Immunostaining was performing using: IB4 (IsolectinB4 10 #121412, 10 μg/ml; Life Technologies), ERG-Alexa fluor 647 (Abcam, ab196149, 1:500), GOLPH4 (#ab28049, 1:400; abcam) and DAPI (#D1306, 1:1,000; Life Technologies). The eyes of P6 pups were prefixed in 4% PFA for 8 min at room temperature. Retinas were dissected, blocked for 30 min at room temperature in blocking buffer (1% fetal bovine serum, 3% BSA, 0.5% Triton X-100, 0.01% Na deoxycholate, 0.02% sodium azide in PBS at pH 7.4) and then incubated with specific antibodies in blocking buffer overnight at 4 C. The next day, retinas were washed and incubated with IB4 and/or ERG (or DAPI) together with the corresponding secondary antibody overnight at 4 C, then washed and post-fixed with 1% PFA and mounted in fluorescent mounting medium (DAKO, USA). High-resolution pictures were acquired using Leica SP8 confocal microscope with a Leica spectral detection system (Leica TCS SP8 detector), and the Leica application suite advanced fluorescence software. After segmenting each channel corresponding to the Golgi and nuclear staining, the centroid of each organelle was determined and a vector connecting the centre of the nucleus to the centre of its corresponding Golgi apparatus was drawn. The Golgi-nucleus assignment was done automatically minimizing the distance between all the possible couples. The polarity of each cell was defined as the angle between the vector and the scratch line. An angular histogram showing the angle distribution was then generated. Circular statistics were performed using a cell polarization toolbox (https://github.com/batho2n/ec_polarization).

To measure the distribution of ECs within each experiments, we created graphs representing the vascular plexus in each experiment by skeletonising images of IB4 staining *via* the software tool PolNet using methods previously described (Bernabeu et al., 2018). Images were prepared and processed using Fiji (Schindelin et al., 2012), GIMP (GNU Image Manipulation Program, 2.10.12), and MATLAB (MathWorks, R2020a). Graphs were simplified to only include edges which formed an angle greater than 30°, and the capillary plexus was manually segmented out from the arteries and veins. Nuclei positions were calculated by binarizing images of Erg staining. Each nuclei was assigned to a vessel edge based on the minimum distance from the centroid to edge midpoints, yielding a dataset of cell number amongst vessel edges for each experiment. We then calculated the max cell number and variance in cell number amongst vessel edges and normalised by the mean cell number for each experiment, providing measures that were comparable to the features MC and VC in the computational data (see feature definition below). We also measured the mean edge length and total number of cells in each experiment. Statistical significance between measurements in Alk1 iKO plexuses vs. Control was assessed *via* Welch’s *t*-test (*α* = 0.05). Five capillary plexuses were obtained for each phenotype, and raw image data used in each can be found in Supplementary Figure S1 for the Control experiments and Supplementary Figure S2 for the Alk1 iKO experiments.

### Flow Within an Idealised Capillary Plexus

Our simulations were performed within an idealised capillary bed constructed of vessels assembled into a “honeycomb” pattern, each with equal length (*α*) and bifurcating at right angles. Each vessel was discretised into cylindrical segments representing the vessel lumen, the surfaces of which were seeded with an initial number of EC agents ( $N_{0}$ ) to represent the endothelium. These EC agents were elliptical in shape with an undeformed length (i.e., major axis) of 2*A* and undeformed width (i.e., minor axis) of 2*B* (see further description of agents below). Luminal segments, each identified by an uppercase subindex *J*, had the same length of an EC ( $Z_{J}=2A$ ) and a diameter defined by wrapping the number of ECs currently occupying the segment, $N_{J}$ , into the circumference of a circle such that,

$$D_{J}=\frac{N_{J}2B}{\pi }$$ (1)

The simulations in this study were performed within an 3 × 3 honeycomb plexus with vessel length *α* = 50 μm, each discretised into five luminal segments initially seeded with $N_{0}$ = 4 cells of length 10 μm and width 5 μm. These values for vessel and EC dimensions come from estimates taken from images of capillary beds in mouse retina models of angiogenic remodelling (Franco et al., 2015).

Vessel lumens are filled with a pressurised fluid (blood), with pressure P stored at each connection point between luminal segment (i.e., nodes). Nodes could vary in degree depending on how many segments they connect: degree one nodes are associated with only one segment and are found at the boundary of the domain, degree two nodes are associated with serial connections between two adjacent segments, and degree three nodes are associated with three segments assembled into a bifurcation. Pressure boundary conditions were prescribed at all degree one nodes ( $P_{\text{in}}$ at the inlets and $P_{\text{out}}$ at the outlets) as to produce forward flow (i.e., $P_{\text{in}}>P_{\text{out}}$ ). We then solve for the unknown nodal pressures and thus flow by assembling a system of equations based on the conservation of mass. The conductance of flow G (i.e., the amount of flow generated from a given pressure gradient) for a segment *J* was calculated as,

$$G_{J}=\frac{\pi D_{J}^{4}}{128\mu Z_{J}},$$ (2)

with *μ* an estimate of the dynamic viscosity of blood. Flow Q through segment *J* is given by the conductance and the pressure difference between the two nodes associated with the segment,

$$Q_{J}=-G_{J}\Delta P_{\mathrm{J.}}$$ (3)

We assigned a conservation of mass equation to each node depending on the node degree. For degree two nodes connecting segments *J* and *K*,

$$Q_{K}-Q_{J}=-G_{K}\Delta P_{K}+G_{J}\Delta P_{J}=0.$$ (4)

For degree three nodes connecting segments *J*, *K*, and *L*,

$$Q_{L}-Q_{K}-Q_{J}=-G_{L}\Delta P_{L}+G_{K}\Delta P_{K}+G_{J}\Delta P_{J}=0.$$ (5)

The final system of equations can be arranged into matrix-vector notation as

[G]⋅{p}={b} (6)

where [G] is the coefficient matrix assembled from vessel connectivity and conductance, {b} is the solution array which contains the boundary conditions, and {p} is the array containing pressure values stored at nodes. We can solve for the nodal pressures by inverting the matrix [G] *via* the *numpy. linalg.solve* function, part of the *SciPy Python* library. Once we have determined pressure throughout the network, we can calculate flow *via* Eq. 3. Vessels with a magnitude of flow less than $10^{-5}\;\mu l\text{/hr}$ were designated as “no flow” for the purpose of quantifying perfusion loss (see feature definitions below).

### Overlapping Ellipses and Transmission Forces

We simulated the endothelium as overlapping ellipses confined to the cylindrical surfaces of luminal segments. Our methods are an updated version of the overlapping spheres method previously used to model tissue mechanics (Pathmanathan et al., 2009) combined with our previous agent-based model of flow-mediated migration (Edgar et al., 2021). Agents representing ECs consisted of nested ellipses: one inner and one outer. The inner ellipse (semi-major axis *A*, semi-minor axis *B*) represents the undeformed “stress-free” configuration of the cell. The outer ellipse (semi-major axis ΓA , semi-minor axis ΓB ) represents the “yield surface” of cellular stretch, with Γ the stretch ratio at which cells will release their junctional connections with neighbours; this can be interpreted as the dimensions a cell will stretch to before yield. We chose an ellipitcal representation of ECs based on the work by Tabibian et al. who found elongation of ECs to be a critical aspect in simulating angiogenic remodelling (Tabibian et al., 2020).

Fields associated with an agent are indicated by a lowercase subindex, e.g., *i*. The location of each EC agent (residing within luminal segment *J* with length $Z_{J}$ and radius $R_{J}=D_{J}/2$ ) is given by its position $x_{i}$ with longitudinal component *z* and circumferential component *c*,

$$\left\{x_{i}\right\}=\left\{\begin{array}{c} z\\ c \end{array}\right\},\;z\in \left[0,\;Z_{J}\right],\;c\in [0,\;2\pi R_{J}].$$ (7)

The circumferential component can also be expressed as the circumferential angle,

θ=cRJ, θ∈[0, 2π]. (8)

EC agents are also polarised and align their major axis along a polarity vector, ${\hat{p}}_{i}$ , which is defined against the direction of flow within the luminal segment they reside.

EC agents transmit force to one another depending on the overlap of adjacent ellipses. The overlap between cell *i* and neighbouring cell *j*, $\delta_{ij}$ , is calculated from on the distance vector between the 2 cells, $r_{ij}$ (with magnitude $L_{ij}$ and unit vector ${\hat{r}}_{ij}$ ). This distance vector is calculated as the difference in longitudinal and circumferential position between the two cells,

$$\left\{r_{ij}\right\}=\left\{\begin{array}{c} \Delta z\\ \Delta c \end{array}\right\},$$ (9)

and requires consideration of periodicity in circumferential position. Details on how we calculate the distance vector between two cells, $r_{ij}$ can be found in Supplementary Methods S1.

Supplementary Figure S12 provides a schematic of how overlap is calculated between two EC agents. The angles of intersection were defined as the angle formed between $r_{ij}$ and ${\hat{p}}_{i}$ for cell *i* ( $\phi_{ij}$ ), and between $-r_{ij}$ and ${\hat{p}}_{j}$ for cell *j* ( $\phi_{ji}$ ). The radius of intersection is defined as the radius of the ellipse at the intersection angle, with the inner radius of intersection for cell *i* defined as

$$\lambda_{i}=\frac{AB}{\sqrt{A^{2}\sin^{2}\phi_{ij}+B^{2}\cos^{2}\phi_{ij}}}.$$ (10)

Similarly, the inner radius of intersection for cell *j* are defined as

$$\lambda_{j}=\frac{AB}{\sqrt{A^{2}\sin^{2}\phi_{ji}+B^{2}\cos^{2}\phi_{ji}}},$$ (11)

Finally, the overlap of the inner ellipses is calculated as

$$\delta_{ij}=\frac{\lambda_{i}+\lambda_{j}-L_{ij}}{2A},$$ (12)

We also define $\delta_{\text{out}}$ as the outer boundary for overlap,

$$\delta_{\text{out}}=\frac{\left(1-\Gamma \right)(\lambda_{i}+\lambda_{j}).}{2A}$$ (13)

The force transmitted between cells, $f_{ij}$ , is defined in a piecewise fashion depending on the overlap between them,

$$f_{ij}=\{\begin{array}{c} -k_{\text{ext}}\delta_{ij}^{2}{\hat{r}}_{ij}\text{, if }\delta_{ij}\geq 0\;\\ k_{\text{coh}}\delta_{ij}^{2}{\hat{r}}_{ij}\text{, if }\delta_{\text{out}}\leq \delta_{ij}<0.\\ 0\text{, if }\delta_{ij}<\delta_{\text{out}} \end{array}$$ (14)

If the inner ellipses of the agents overlap ( $\delta_{ij}\geq 0$ ) the agents transmit an extrusive force (scaled by $k_{\text{ext}}$ ) to push the agents back to the stress-free configuration. If the outer ellipses of the agents overlap ( $\delta_{\text{out}}\leq \delta_{ij}<0$ ) the agents transmit a cohesive force (scaled by $k_{\text{coh}}$ ) to pull the agents back to the stress-free configuration. The agents are out of range from each other if $\delta_{ij}<\delta_{\text{out}}$ and thus transmit no force. The use of $\delta_{ij}^{2}$ in the definition of the transmission force has several advantages in that $\delta_{ij}^{2}$ is symmetric about the origin, has a global minima at the interface between inner/outer ellipses which is also the transition between extrusion/cohesion, and scales quadratically between 0 and 1. These features stabilise the behaviour of the model and make the related parameters easier to interpret; i.e., $k_{\text{ext}}$ and $k_{\text{coh}}$ set upper bounds on the magnitude of transmission forces in response to overlap. Lastly, in addition to the passive transmission produced by overlap, EC agents also produce an active migration force along their polarity vector (scaled by $k_{\text{mig}}$ ) which causes them to migrate against the direction of flow.

The parameters regulating force transmission ( $k_{\text{ext}}$ , $k_{\text{coh}}$ , and Γ ) can be combined and interpreted as the energy required to either push cells together (against extrusion) or pull cells apart (against cohesion). The work required to push two agents (major axis aligned) together such that they completely overlap is given by,

$$W_{\text{push}}=\int_{0}^{1}k_{\text{ext}}\delta^{2}2Ad\delta =\frac{2Ak_{\text{ext}}}{3},$$ (15)

which scales linearly with respect to the extrusion parameter $k_{\text{ext}}$ . Similarly, the work required to pull the two cells apart is given by,

$$W_{\text{pull}}=\int_{0}^{\delta_{\text{out}}}k_{\text{coh}}\delta^{2}2Ad\delta =\frac{2Ak_{\text{coh}}{\left|1-\Gamma \right|}^{3}}{3}.$$ (16)

The amount of work required for a cell to migrate an arbitrary distance *X* is

$$W_{\text{mig}}=\int_{0}^{X}k_{\text{mig}}dx=k_{\text{mig}}X.$$ (17)

If we choose X=2A/3 we can obtain the normalised work parameters by dividing $W_{\text{push}}$ and $W_{\text{pull}}$ by $W_{\text{mig}}$ ,

$$W_{\text{push}}^{*}=\frac{k_{\text{ext},}}{k_{\text{mig}}}$$ (18)

$$W_{\text{pull}}^{*}=\frac{k_{\text{coh}}{\left|1-\Gamma \right|}^{3}}{k_{\text{mig}}}$$ (19)

Note that the work required to break apart cell cohesion scales linearly with respect to the cohesion parameter $k_{\text{coh}}$ and cubically to the yield stretch, Γ .

### Migration Dynamics

We implemented an overdamped dynamic model of migration to calculate the migration velocity of each EC agent, $v_{i}$ , *via* the balance of a damping force with the sum of passive forces transmitted from neighbouring agents and the active migration force,

$$\eta v_{i}=\sum_{j}f_{ij}+k_{\text{mig}}{\hat{p}}_{i},$$ (15a)

with damping parameter η that relates force to migration speed. The migration force parameter $k_{\text{mig}}$ and damping parameter eta combine to produce the mean migration velocity,

v¯=kmigη. (16a)

In all our simulations, $\bar{v}$ was set to 3.0 μm/h which was the mean migration speed observed in live tracking of migrating ECs in angiogenic remodelling within the zebrafish trunk vasculature (Rosa et al., 2020). This model assumes quasi-static equilibrium at each migration step, implying that all forces sum to zero and the effects of inertia are negligible. Once we have calculated migration velocity, we can update each EC agent to its new position using Euler’s method with time step size Δt ,

$$x_{i}:=x_{i}+v_{i}\Delta t.$$ (17a)

If the longitudinal component *z* of the new position exceeds the bounds (i.e., $z>Z_{L}$ or z<0 ), the agent is moved to the appropriate neighbouring segment (either upstream or downstream). Agents continue migrating across neighbouring segments until they reach a bifurcation: at flow-convergent bifurcations, EC migration paths converge and cells within both child branches simple combine at the parent branch. However, at flow-divergent bifurcations EC migration paths diverge requiring that cells in the parent branch split evenly amongst the two child branches. This splitting is performed based on the circumferential angular position θ of each agent. The child branches are assigned as “left” or “right” depending on the sign of the angle the branch forms with the *x*-axis: if θ≤π the cell migrates into the left branch, otherwise the cell migrates into the right branch. Note that the splitting of cells at bifurcation is done solely based on position, which will result in ECs migrating into branches with no flow/shear stress. Lastly, we prescribed periodic boundary conditions on the cells at the inlets and outlets, such that any cell exciting at an inlet re-enters the plexus as the corresponding outlet; these conditions keep the total number of EC agents within the domain constant.

As agents move to new segments, thy may do so in a way that creates new overlap with current residents, creating spikes in velocity. In order to enforce our assumption of quasi-static equilibrium, we allow the cells to passively intercalate to an “acceleration-free” configuration after each migration step. This involves iteratively displacing the cells based on passive transmission forces only until the set of velocities amongst all cells, ${\left\{v\right\}}_{\text{cells}}$ , satisfies a convergence criterion. For each intercalation iteration *k*, velocity is calculated as

$$\eta v_{i}=\sum_{j}f_{ij,}$$ (18a)

and the system is deemed converged when the L2 norm of the velocity difference drops below a tolerance parameter $\varepsilon_{\mathrm{int}}$ ,

$$\left\|{\left\{v\right\}}_{\text{cells}}^{k}-{\left\{v\right\}}_{\text{cells}}^{k-1}\right\|<\varepsilon_{\mathrm{int}.}$$ (19a)

Information of the parameters used in our study in Table 1.

**TABLE 1:** Description of parameters and prescribed values.

Symbol	Description	Values	Units
α	Vessel edge length	50	μm
$N_{0}$	Initial cell number per segment	4	-
$P_{\text{in}}$	Inlet pressure boundary condition	1,296	kg/μm-hr^2^
$P_{\text{out}}$	Outlet pressure boundary condition	0	kg/μm-hr^2^
μ	Dynamic viscosity of blood	1.26e-5	kg/μm-hr
*A*	Semi-major axis of EC agents	5	μm
*B*	Semi-minor axis of EC agents	2.5	μm
η	Migration dampening parameter	1, 3, 5	kg/hr
$k_{\text{ext}}$	Extrusive force scaling parameter	*η,* 3*η,* 9*η*, 15*η*	kg- μm/hr^2^
$k_{\text{coh}}$	Cohesive force scaling parameter	0, (1/3)*η,* (2/3)*η, η*	kg- μm/hr^2^
Γ	Yield stretch	1.25, 1.5, 1.75	-
$k_{\text{mig}}$	Migration force scaling parameter	3*η*	kg- μm/hr^2^
$\bar{v}$	Mean migration velocity	3	μm/hr
Δt	Euler time step size	0.5	Hr
$T_{\text{end}}$	End time	96	Hr
$\varepsilon_{\mathrm{int}}$	Intercalation convergence tolerance	2e-2	μm/hr

### Network-Level Features Indicating Shunt Formation

We calculated several network-level features indicating the status of the vascular plexus to indicate the emergence of shunts during remodelling. These shunt features are based on summary statistics extracted from the set of all vessels, the set of all cells, or the set of all time points. For the sake of simplicity, the features will be defined algorithmically within this section.

The first set of features indicate the overall level of stress stored within the endothelial tissue by measuring overlap amongst the EC agents:

• Mean Cell Compression (CC)—each cell stores the sum of all negative overlap it experiences at each time point; take the mean amongst all cells for every time point; take the time-averaged across all points in time.
• Mean Cell Tension (CT)—each cell stores the sum of all positive overlap it experiences at each time point; take the mean amongst all cells for every time point; take the time-averaged across all points in time.

The next set of features indicate cell aggregation within the plexus. These features will be low if the cells remain evenly distributed throughout the plexus, and will increase if the distribution becomes skewed:

• Max Cells amongst vessels (MC)—take the max number of cells amongst all vessels (i.e., size of the largest vessel) at each time point; take the time-averaged value across time; divide by mean number of cells amongst vessels.
• Variance in Cells amongst vessels (VC)—take the variance in cell number across all vessels; take the time-averaged value across time; divide by the mean number of cells amongst vessels.

Note that with these features, the mean number of cells amongst vessels always equals $N_{0}$ due to the periodic boundary conditions (total number of cells divided by total number of vessels equals $N_{0}$ ).

The last set of features indicate the level of flow disruption occurring during remodelling. These features either measure the amount of time vessels experience with disturbed flow (either flow reversal or flow loss), and the amount of perfusion loss within the plexus. Perfusion loss was calculated as the percentage of all possible flow paths (34 flow paths in total for the 3 × 3 honeycomb plexus) in which flow dropped to zero at some point along the path:

• Time Flow Reversed (FR)—amongst all vessels, if flow in a vessel is negative when it was positive in the previous time step, add Δt to TFR; divide by $T_{\text{end}}$ .
• Perfusion Loss (PL)—for each time point, calculate the percentage of flow paths with flow loss; take the time-average across time points.

We ran our model with 50 different random seed numbers (randomising the initial position of agents each time) at various parameter values ( $k_{\text{ext}}$ , $k_{\text{coh}}$ , Γ , η , and Δt ; Table 1 for range of values) and collected populations of these features in each case. We also calculated Pearson’s correlation coefficient (using the MATLAB function *corrcoef*) between each of the features and the parameters, and between the features themselves.

### Computational Fluid Dynamics of Flow Reversals in the Honeycomb Plexus

We simulated the collapse of a single vessel within an ideal 3D honeycomb (3 × 3) vascular plexus (hence referred to as Vessel A, indicated by black arrows in the inset of Figure 6A) to inspect fluid behaviour upon flow reversals and determine the amount of lumen reduction required to generate a flow reversal.

Fluid domain meshes discretising the ideal vascular networks were generated using the Vascular Modelling Toolkit in *Python* (VMTK http://www.vmtk.org/) (Izzo et al., 2018), *via* the following steps: 1) First we constructed a 2D honeycomb network of nodes with the assigned vessel radius defined at each node. The radius of an open vessel (a fully patent vessel) was $r_{open}=4\;\mu \text{m}$ . Each vessel had a length of 2 μm between bifurcations and a bifurcation angle of π/2 . 2) We used the *vmtkcenterlinemodeller* tool to generate a 3D rendering of each 2D honeycomb network and 3) then the *vmtksurfaceremeshing* tool to produce a closed structured surface mesh. 4) To remove the caps over inlets and outlets, we used the *vtkClipPolyData* tool to produce a series of open structured meshes of an idealised vascular network.

We used the HemeLB software (Mazzeo and Coveney, 2008) to simulate blood flow through each network in the sequence of static meshes, allowing us to examine the dynamic changes in the fluid flow as Vessel A collapsed. HemeLB is a high-performance lattice-Boltzmann solver developed to simulate blood flow through complex static 3D vascular geometries. The lattice-Boltzmann method is built on microscopic models and discretised mesoscopic kinetic equations to recreate the dynamics of incompressible fluid flow on a regular lattice. The fluid density is calculated at each discrete point in the lattice, and update through a statistical streaming and collision process at discrete time steps with discrete velocities (Krüger et al., 2017).

We used a D3Q15 lattice, the Bhatnagar-Gross-Krook collision operator (see e.g., (Succi, 2001) for details) and the simple bound-back boundary condition. To avoid successive simulations using the same number of voxels across Vessel A, we needed to ensure the voxel size was less than the radial decrease in Vessel A between successive networks. As such, we discretised each network using a voxel size of $v_{x}=1.95\times 10^{-3}\;\mu \text{m}$ , providing 41 voxels across the diameter of a fully patent vessel, and a time step of $dt=9.52\times 10^{-8}\text{ s}$ . This large number of voxels satisfied the requirement of a minimum of 15 voxels across the diameter for stable flow using HemeLB (Bernabeu et al., 2014). Simulations ran for 10,000 time steps, ensuring fully developed flow in each network. Flow through each network was pressure driven. We used a pressure of 100 Pa at each inlet, and a pressure of 0 Pa at each outlet.

The radius of Vessel A was reduced in intervals of $∼0.1r_{open}$ (or 10%). The radius of Vessel A was decreased in intervals by removing the outer layer of voxels from the vessel. Owing to the Cartesian discretisation used in HemeLB the effective radius of the vessel, $r_{eff}$ , was lower than that prescribed during the mesh development. Lumen reduction of Vessel A, $A^{*}$ , was calculated as the ratio $r_{eff}/r_{open}$ . We examined the flow though the two diagonal feeding vessels as Vessel A progressively collapsed using Paraview (Hansen and Johnson, 2005; Ayachit, 2015). To do this we calculated the normalised flow $Q^{*}$ through a cross-section defined by a perpendicular plane bisecting the vessel normalised by the flow through the upper inlet vessel, which was calculated in the same manner. The normalised rotation of the flow $R^{*}$ was calculated as the mean absolute rotation through each vessel, and normalised to the mean absolute inlet rotation. Here the rotation is scalar defined by the rotation angle of each fluid particle along a streamline.

## Data Availability

All software and analysis tools were written in Python 3.6.8 and MATLAB R2020a. Source code (including the random seed numbers used to generate the data in this study) is available on Github at: https://github.com/ltedgar-ed/ABM_flow_migrate_angio_v2_overlapping_ellipses_release.
