# Supplementary material for: Traffic Patterns of the Migrating Endothelium: How Force Transmission Regulates Vascular Malformation and Functional Shunting During Angiogenic Remodelling
Source: Front Cell Dev Biol. 2022 May 19;10:840066. doi: 10.3389/fcell.2022.840066 (PMC9160721; doi:10.3389/fcell.2022.840066)
Supplement: Supplementary file 2 [file DataSheet1.pdf]

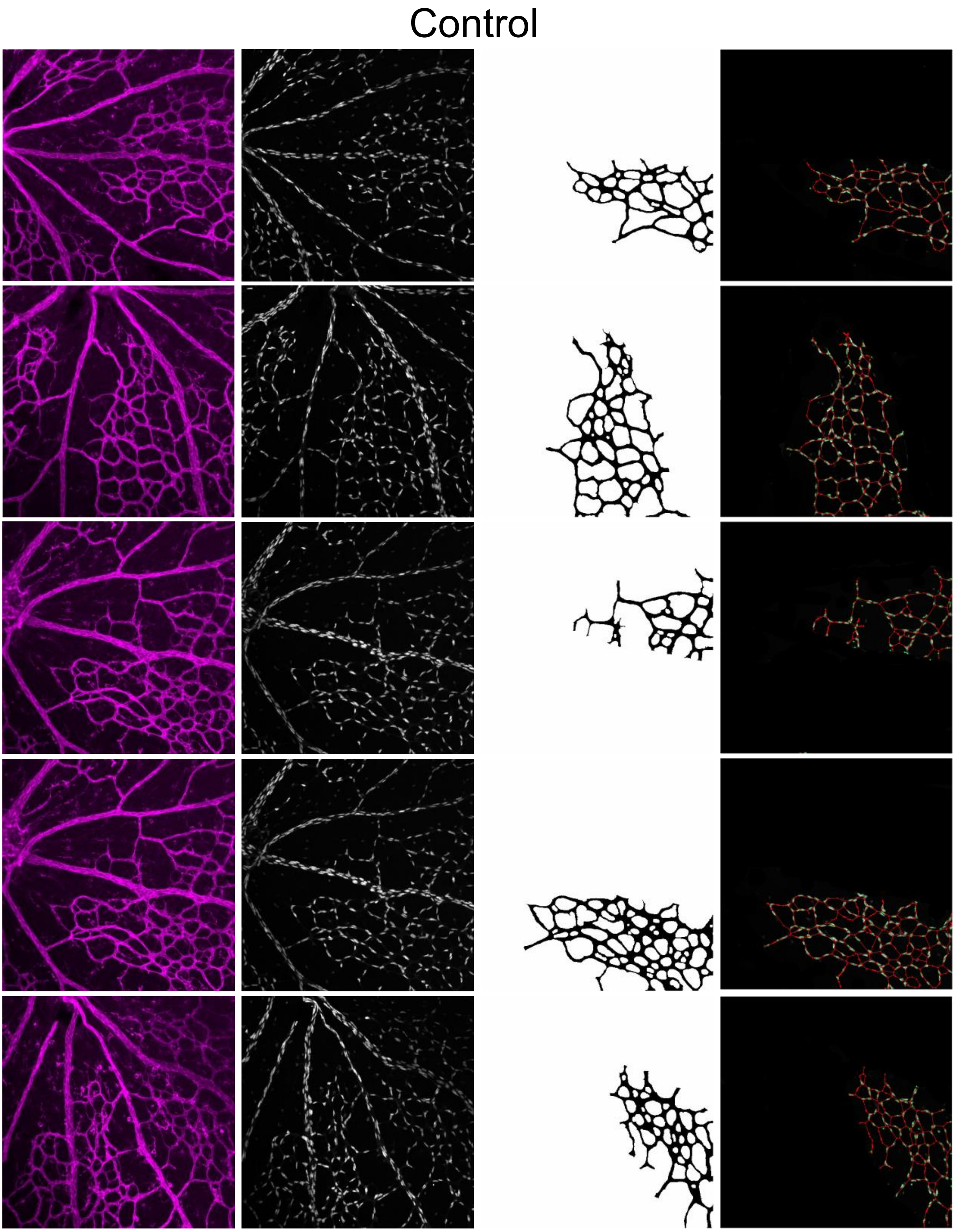

Supplementary Figure 1. Data from Control mouse retina experiments: (1st column) IB4 labelling vessel lumens, (2nd column) ERG labelling EC nuclei, (3rd column) segmented capillary plexus, (4th column) skeletonised vessel wedge (red) and nuclei position (green).

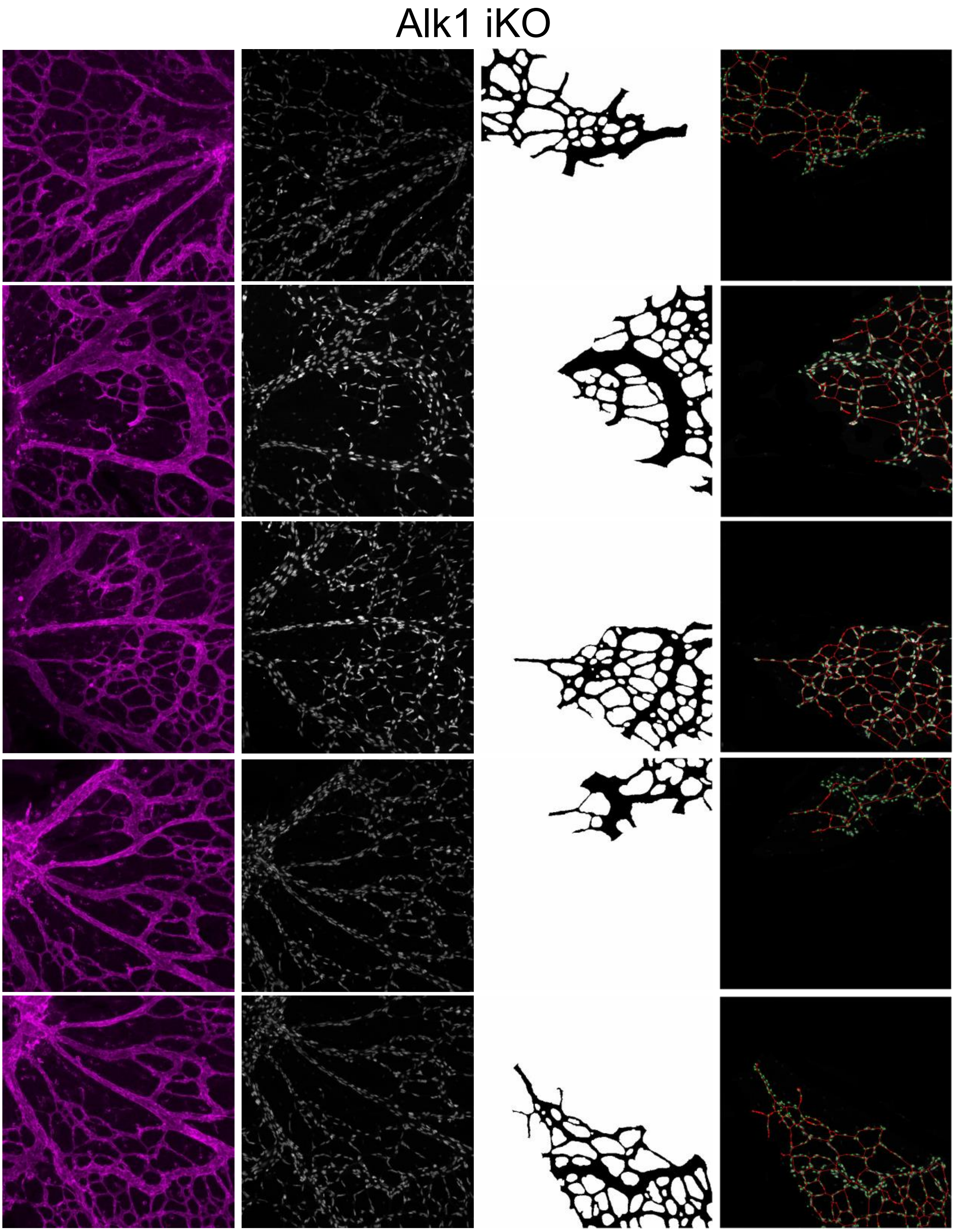

Supplementary Figure 2. Data from Alk1 KO mouse retina experiments: (1st column) IB4 labelling vessel lumens, (2nd column) ERG labelling EC nuclei, (3rd column) segmented capillary plexus, (4th column) skeletonised vessel wedge (red) and nuclei position (green).

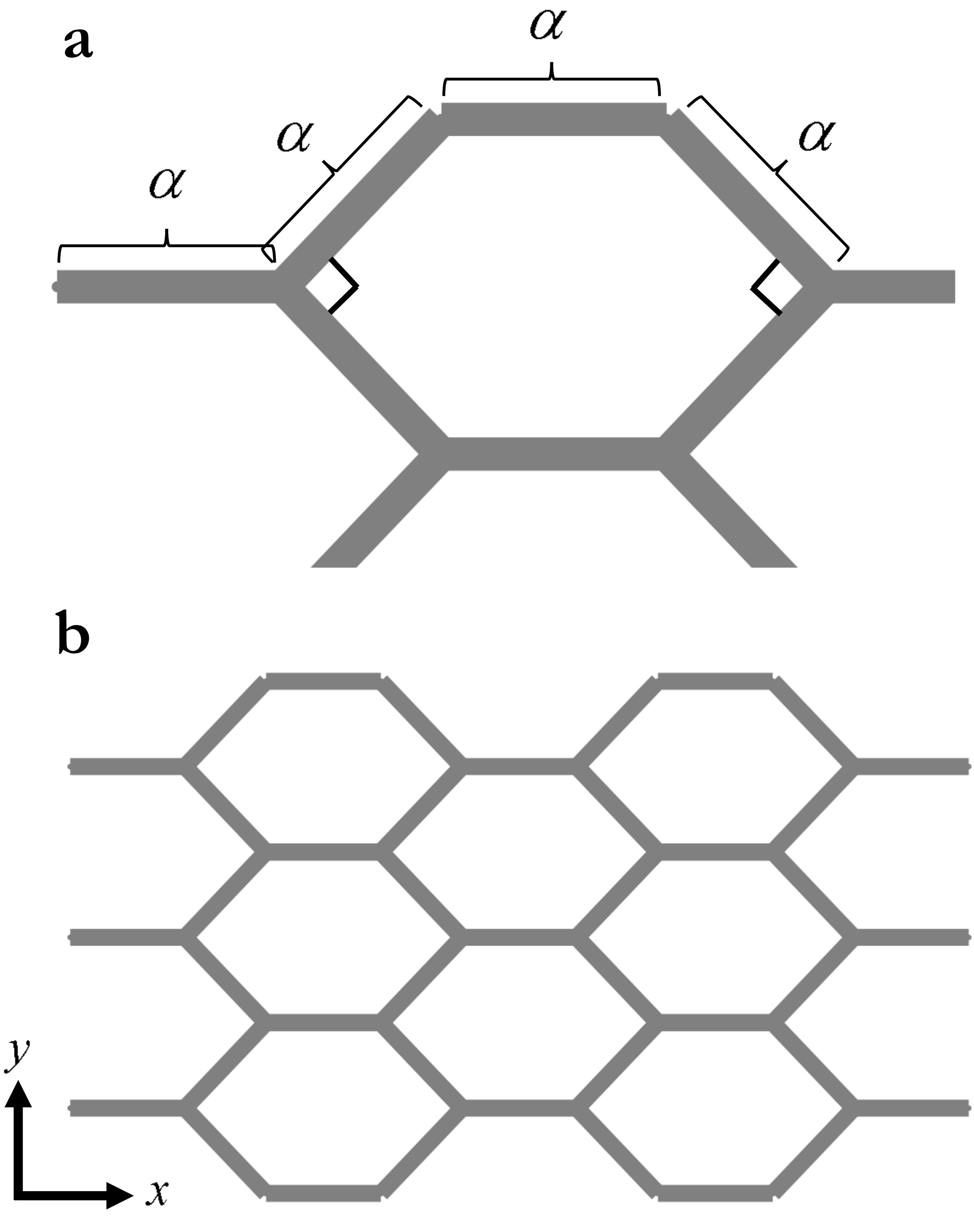

Supplementary Figure 3. The idealised capillary plexus domain. (a) The “honeycomb” vessel configuration with edges all of equal length α and bifurcations formed at right angles. (b) We simulated flow-mediated migration and remodelling within a 3x3 plexus domain.

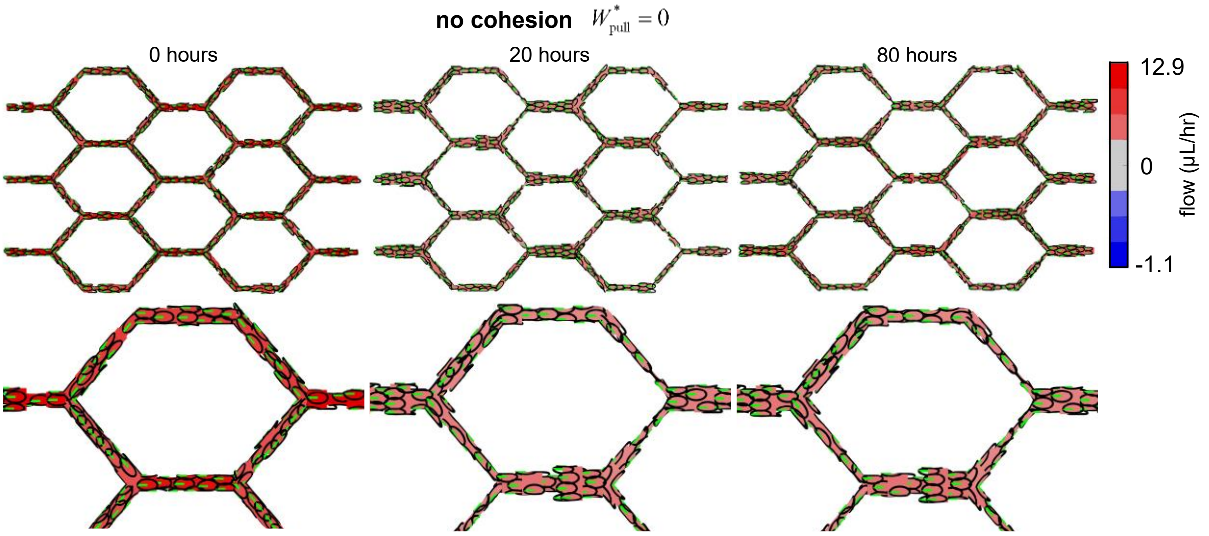

Supplementary Figure 4. Extrusive forces without cohesion maintain an even distribution of cells throughout the plexus during remodelling. In this example simulation without cohesion ($W^*_{\text{pull}} = 0$), the whole plexus domain in the top row and zoomed in region of a single honeycomb cell on the bottom row.

Supplementary Video 1. Simulation of remodelling in a cell population uniform polarised against flow without cohesion ($W^*_{\text{push}} = 3$; $W^*_{\text{pull}} = 0$). Forward flow is indicated by red, reversed flow by blue, and no flow in grey. Note that any flow reversals that develop are quickly resolved and cells remain evenly distributed throughout the plexus through the entirety of the simulation.

Supplementary Video 2. Simulation of remodelling in a cell population uniform polarised against flow with high cohesion ($W^*_{\text{push}} = 3$; $W^*_{\text{pull}} = 3/72$). Note that flow reversals persist for long periods of time and cells pool into large aggregates which deplete other regions of the plexus of cells and perfusion.

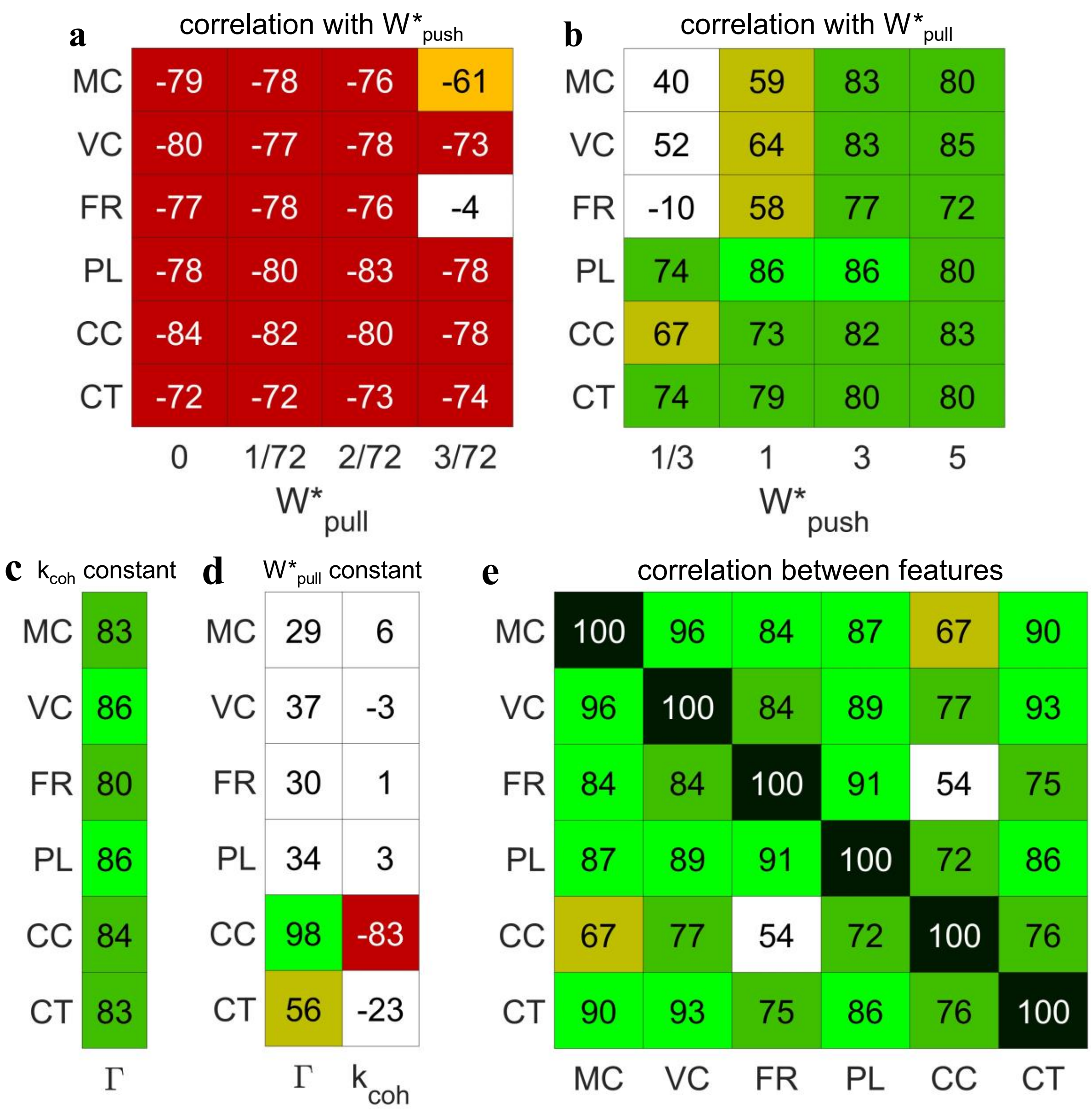

Supplementary Figure 5. Correlation analysis between network features and force transmission parameters. (a) Shunt features were negatively correlated with increases in W^*_{push}, demonstrating the stabilising effect of extrusion. (b) Shunt features tended to increase with W^*_{pull} (i.e., positive correlation) demonstrating the disruptive effect cohesion produces during remodelling. (c) Increasing Γ whilst holding k_{coh} increases W^*_{pull} and promotes shunt formation (as seen in positive correlation with shunt features). (d) However, changing k_{coh} and Γ whilst keeping W^*_{pull} constant produced little to no correlation in shunt features within the range of these parameters that we analysed. (e) Intra-feature correlation revealed that most of our shunt features correlated strongly with each other, with the exception of Cell Compression which played a reduced role during shunt formation.

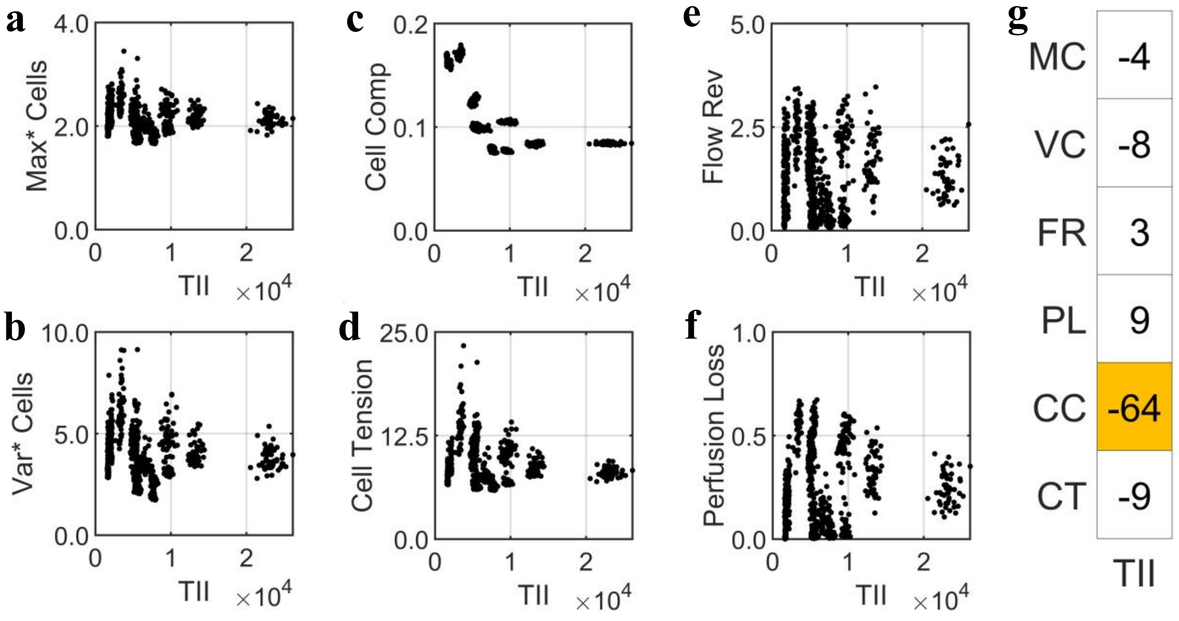

Supplementary Figure 6. Total Intercalation Iterations (TII) during each simulation played no role in shunt formation. Network shunt features indicating: (a) Max cells amongst vessels, (b) Variance in cells amongst vessels, (c) Mean cell compression, (d) Mean cell tension, (e) Time with reversed flow, and (f) perfusion loss vs. Total Intercalation Iterations (i.e., total amount of iterations spent in the intercalation routine during the simulation). (g) We found no correlation between our shunt features and TII, apart from Mean cell compression which showed a weak negative correlation.

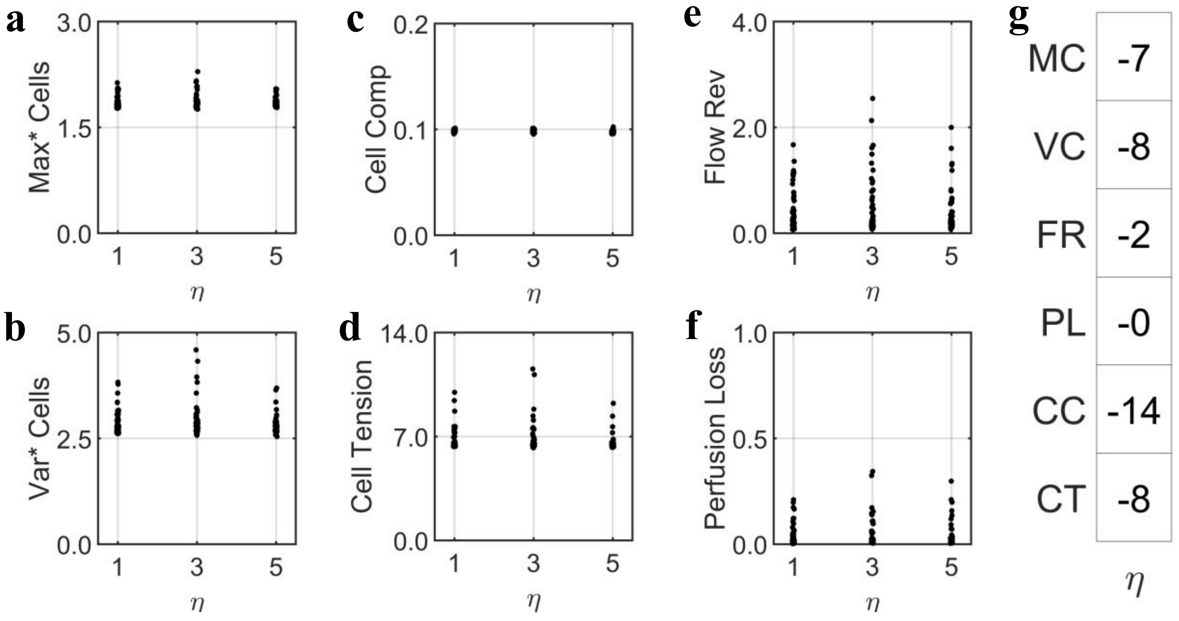

Supplementary Figure 7. Shunt formation was not a function of the dissipative coefficient *η*. No obvious trend was found between our shunt features indicating: (a) Max cells amongst vessels, (b) Variance in cells amongst vessels, (c) Mean cell compression, (d) Mean cell tension, (e) Time with reversed flow, and (f) perfusion loss vs. η. (g) We found no correlation between shunt features and η.

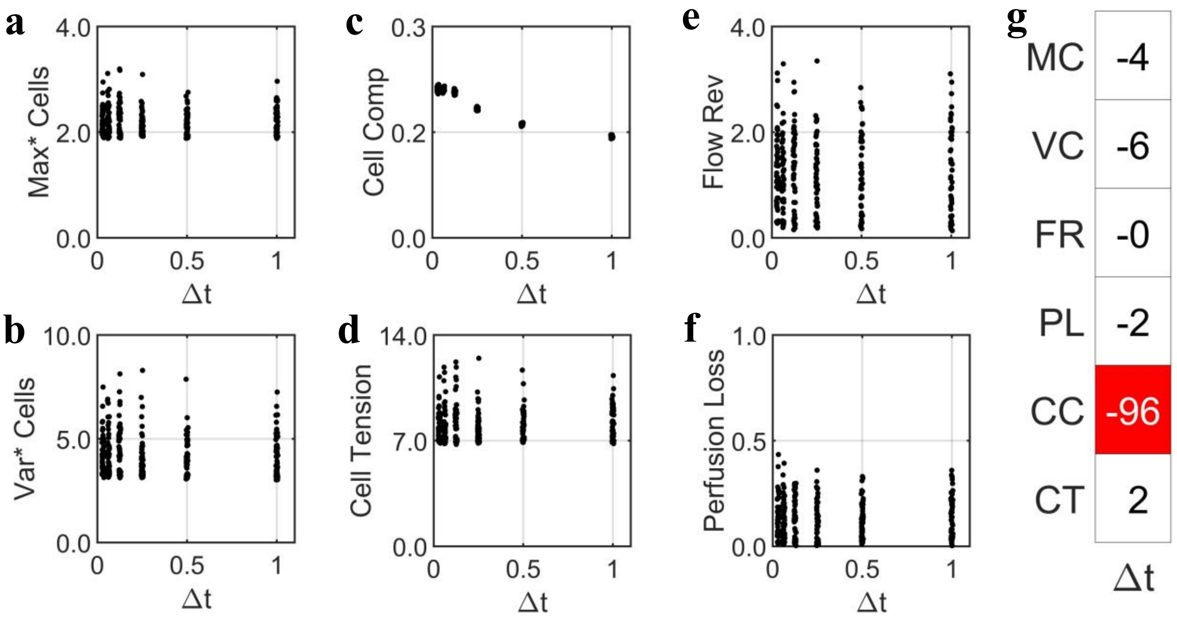

Supplementary Figure 8. Changing the Euler time step size (Δt) had no effect on shunt formation. No obvious trend was found between our shunt features indicating: (a) Max cells amongst vessels, (b) Variance in cells amongst vessels, (c) Mean cell compression, (d) Mean cell tension, (e) Time with reversed flow, and (f) perfusion loss vs. Δt , the time step size in the Euler scheme used to update cell position during migration. (g) We found no correlation between our shunt features and Δt other than a strong correlation with Mean Cell Compression, although the magnitude of changes in were small.

Supplementary Video 3. Simulation of migration of a mixed polarity population (50/50 with/against flow) at low extrusion ($W^*_{\text{push}} = 1/3$; $W^*_{\text{pull}} = 0$). At low levels of extrusion, opposingly polarised cells were able to pass by each other effectively and disruption of the plexus during migration was minimal.

Supplementary Video 4. Simulation of migration of a mixed polarity population (50/50 with/against flow) at high extrusion ($W^*_{\text{push}} = 3$; $W^*_{\text{pull}} = 0$). At high extrusion, opposingly polarised cells found it exceedingly difficult to pass by each other, leading to impaired migration, traffic jams, and disruption of the vascular plexus.

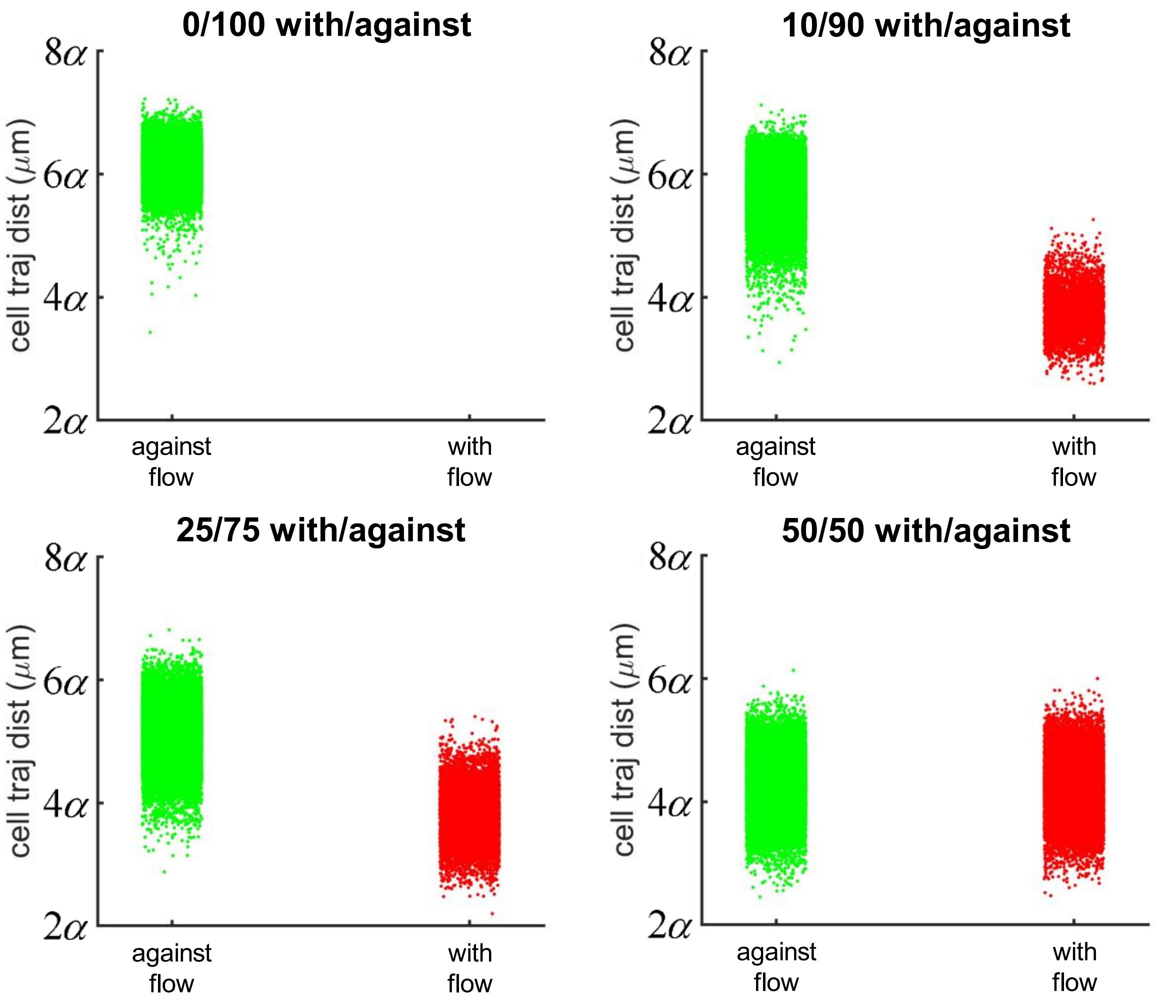

Supplementary Figure 8. Opposing cells become "short" migrators in mixed polarity populations. Cell trajectory distance plotted for all cells polarised against flow (green) and with flow (red). Cells can be classified as "far" migrators with a high value of cell trajectory distance travelled during migration, or as "short" migrators with a lower value travelled. (upper left) All cells in a uniformly polarised population (0/100 with/against flow) were "far" migrators. (upper right) At a mixture of 10/90 with/against flow, cells travelling with flow travelled shorter distances during migration; cells travelling against flow also travelled slightly less. (lower left) At a mixture of 25/75 with/against flow, cells polarised against flow travelled even shorter distances as they experienced further interference from cells polarised with flow. (lower right) Lastly, a mixture of 50/50 with/against flow resulted in all cells travelling short distances during migration as they experienced similar levels of interference from opposingly polarised neighbours.

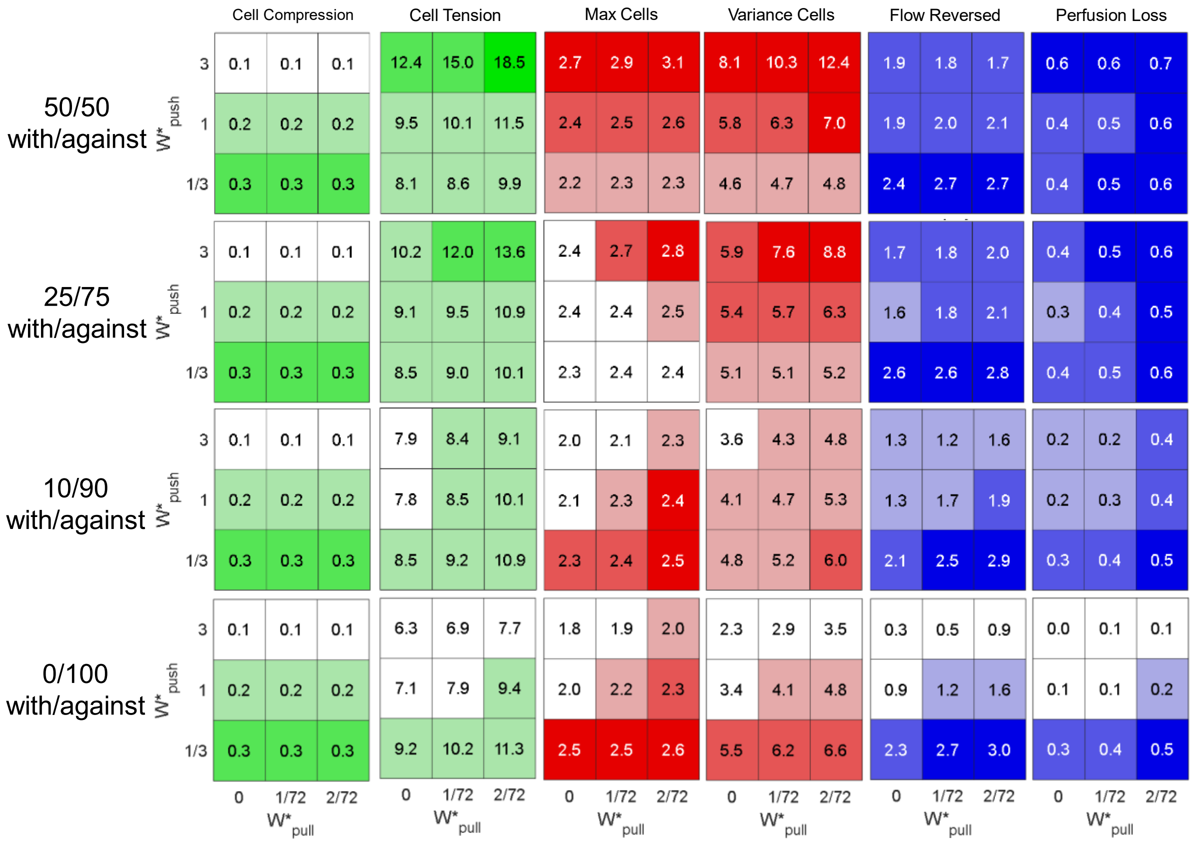

Supplementary Figure 9. Extrusion forces become a destabilising factor in mixed polarity populations. Mean values of network features indicating cell stress (green), cell distribution (red), and flow disruption (blue) are shown for various levels of W^*_{push} and W^*_{pull} for different mixtures of polarity with/against flow. (bottom row) For uniformly polarised populations against flow, increased extrusion tended to decrease/stabilise all features while increased cohesion tended to increase/destabilise. (2nd row up) Adding a small percentage of opposingly polarised cells (10/90 with/against) resulted in slightly increased values amongst all features but maintained the same trend as before. (3rd row up) At a larger percentage of opposingly polarised cells (25/75 with/against), all feature values were higher and network features started to exhibit an opposite trend in which increased extrusion resulted is larger amounts of Cell Tension, uneven distribution of cells, and increased Perfusion Loss. (top row) At a 50/50 mixture of polarisation with/again flow, extrusion tended to increase all features with the exception of Cell Compression and Flow Reversed. Increased cohesion tended to increase features even further.

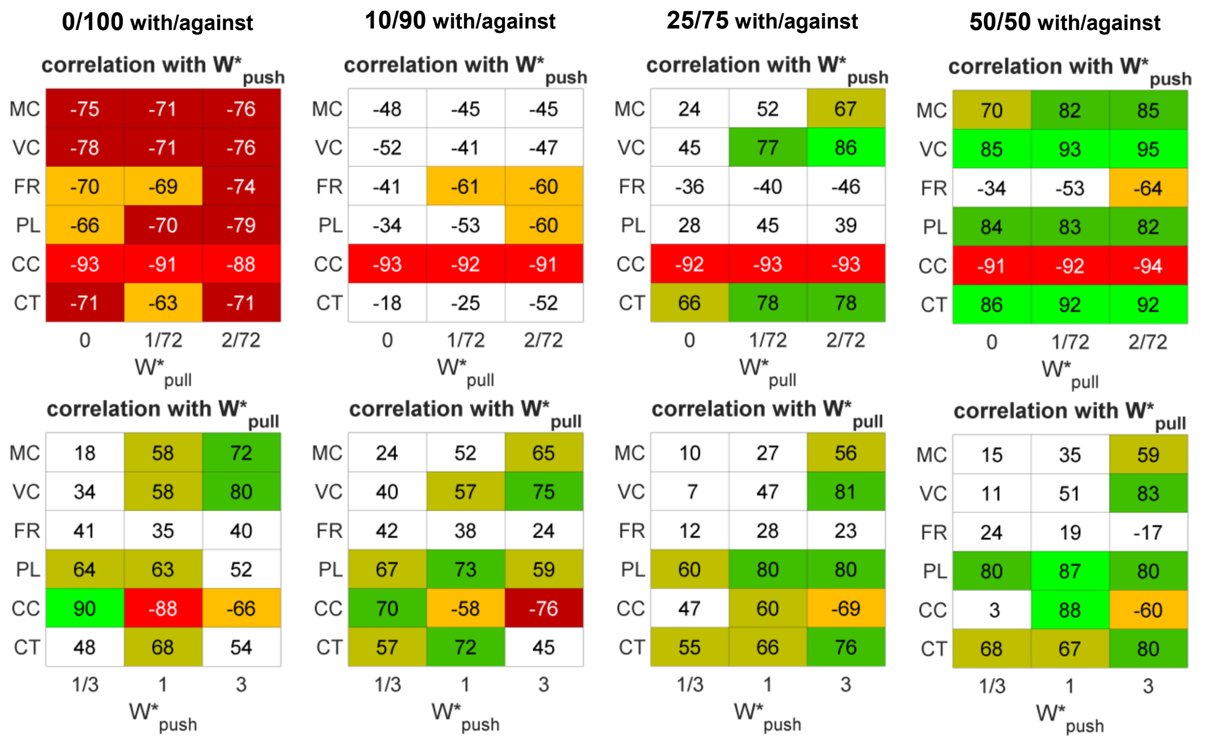

Supplementary Figure 10. Network features switch from negative correlation with extrusion to positive, indicating the change in role of extrusion in mixed polarity populations. Correlation analysis with network features with W^*_{push} at various levels of W^*_{pull} (top row), and with W^*_{pull} at various levels of W^*_{push} (bottom row). In a uniformly polarised population (0/100 with/against flow), features tended to decrease while adding extrusion (seen in orange/red) and increase while adding cohesion (seen in yellow/green). (top row) As we increased the amount of opposingly polarised cells to the population, the correlation with W^*_{push} gradually shifted from negative to positive, indicating the change in role of extrusion forces from stabilisation to destabilisation within mixed polarity population. (bottom row) Network features remained positively correlated with cohesive force, and these correlation tended to increase as we increased the percentage of opposingly polarised cells within the population.

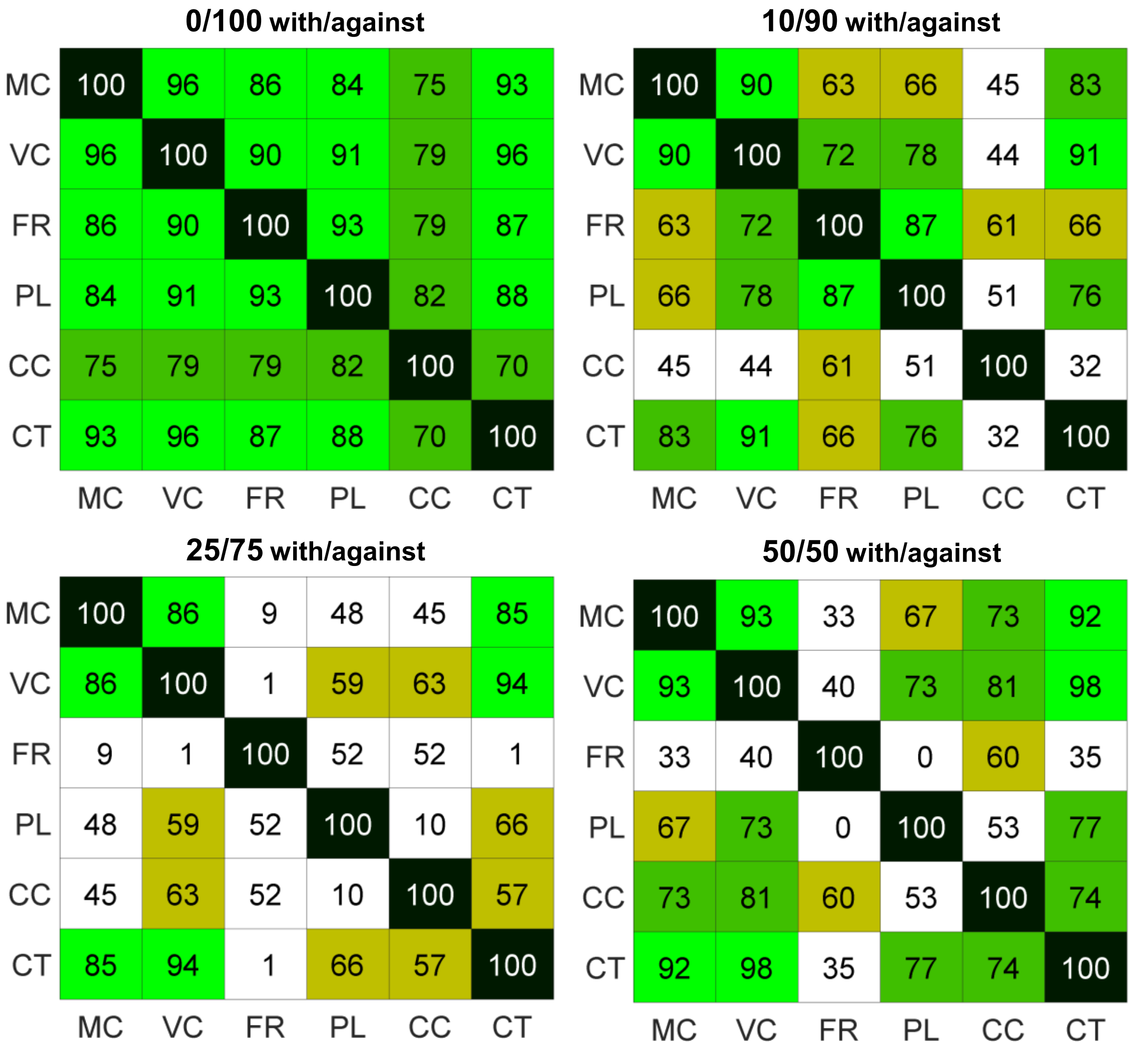

Supplementary Figure 11. Intra-feature correlation between features changes with percentage of opposingly polarised cells. Intra-feature correlation between network features at various levels of mixed polarity with/against flow: 0/100 (upper left), 10/90 (upper right), 25/75 (lower left), and 50/50 (lower right). While all features correlated strongly with each other in the case of uniformly polarised populations, this correlation weakened for some features as the level of mixing increased. In particular, the correlation between Flow Reversals and all other features decreased dramatically as mixing increased, indicating that flow reversals and the resulting directional changes in migration were not associated with vascular malformation and instability in mixed polarity populations.

Supplementary Methods 1. Calculating the distance between agents residing within the surfaces of cylindrical luminal segments.

Distance between two points within the same cylindrical surface

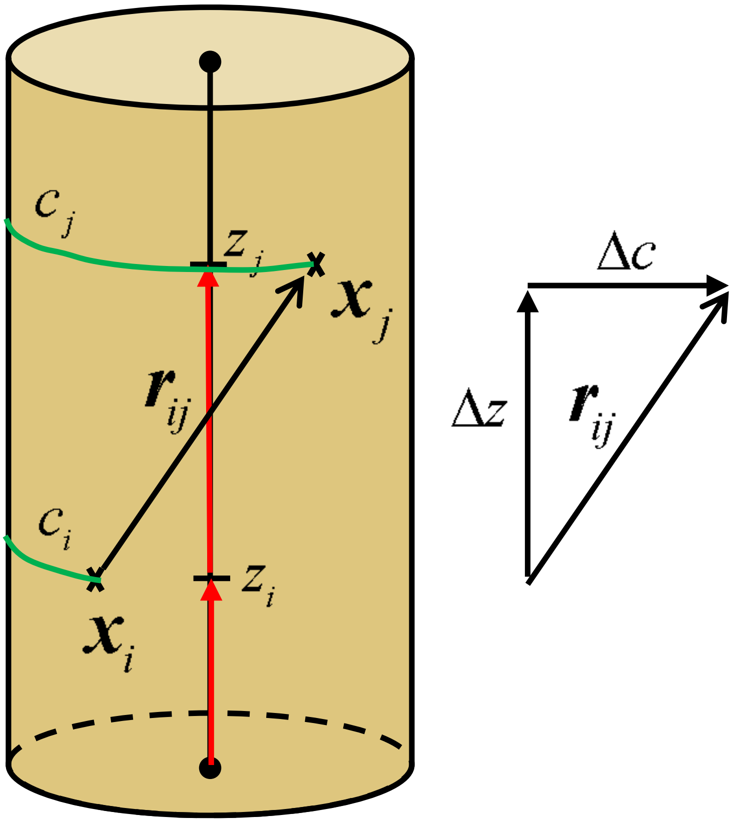

Any point along the circumferential surface of a cylinder can be expressed as a longitudinal position z and a position along the circumference, c. For a cylinder of length Z and radius R,

$$z \in [0, Z],$$

$$c \in [0, 2\pi R].$$

Note that in traditional cylindrical coordinates this is akin to holding the radial position r constant at $r = R$. Furthermore, the circumferential position is just a scaling of the cylindrical angle θ by a constant,

$$c = R\theta, \ \theta \in [0, 2\pi].$$

Additionally, we will define a natural coordinate system for the cylinder surface,

$$\xi = \frac{z}{Z}, \ \xi \in [0,1],$$

$$\zeta = \frac{c}{2\pi R} = \frac{\theta}{2\pi}, \ \zeta \in [0,1].$$

Consider two points within the surface of the cylinder, $\boldsymbol{x}_i$ and $\boldsymbol{x}_j$, with positions

$$\{x_i\} = \begin{Bmatrix} z_i \\ c_i \end{Bmatrix} = \begin{Bmatrix} z_i \\ R\theta_i \end{Bmatrix} = \begin{Bmatrix} Z\xi_i \\ 2\pi R\zeta_i \end{Bmatrix},$$

$$\{x_j\} = \begin{Bmatrix} z_j \\ c_j \end{Bmatrix} = \begin{Bmatrix} z_j \\ R\theta_j \end{Bmatrix} = \begin{Bmatrix} Z\xi_j \\ 2\pi R\zeta_j \end{Bmatrix}.$$

The distance between these two points along the surface of the cylinder is given by the magnitude of the vector $\boldsymbol{r}_{ij}$,

$$\boldsymbol{r}_{ij} = \boldsymbol{x}_j - \boldsymbol{x}_i = L_{ij}\hat{\boldsymbol{r}}_{ij},$$

The distance vector $\boldsymbol{r}_{ij}$ can be expressed as a change in the longitudinal and circumferential coordinates z and c, or change in z and the angle θ,

$$\{r_{ij}\} = \begin{Bmatrix} \Delta z \\ \Delta c \end{Bmatrix}.$$

The change in the longitude z has a straight-forward definition,

$$\Delta z = z_j - z_i = Z\left(\xi_j - \xi_i\right)$$

There are always two equally valid paths to travel along the circumference from $\boldsymbol{x}_i$ to $\boldsymbol{x}_j$ due to the symmetry of the circumference (either by increasing θ or decreasing θ). In our application we will always consider the shortest circumferential path by enforcing an upper limit on the difference in angle θ between the two points,

$$|\Delta\theta| \le \pi .$$

The restricts the largest circumferential difference possible between the two points to be half of the circumference, or one half in the natural coordinates,

$$|\Delta c| \le \pi R,$$

$$|\Delta\zeta| \le 0.5 .$$

To obtain these results, we define $\Delta\theta$ as

$$\Delta\theta = \begin{cases} \theta_j - \theta_i, & \text{if } \left|\theta_j - \theta_i\right| \le \pi \\ \left(\theta_j - \theta_i\right) - 2\pi, & \text{if } \left(\theta_j - \theta_i\right) > \pi \\ \left(\theta_j - \theta_i\right) + 2\pi, & \text{if } \left(\theta_j - \theta_i\right) < -\pi \end{cases}, \quad \Delta\theta \in [-\pi, \pi].$$

The corresponding definition of $\Delta\,\zeta$ is

$$\Delta\zeta = \begin{cases} \zeta_j - \zeta_i, & \text{if } \left|\zeta_j - \zeta_i\right| \le 0.5 \\ \left(\zeta_j - \zeta_i\right) - 1, & \text{if } \left(\zeta_j - \zeta_i\right) > 0.5 \\ \left(\zeta_j - \zeta_i\right) + 1, & \text{if } \left(\zeta_j - \zeta_i\right) < -0.5 \end{cases}, \; \Delta\zeta \in [-0.5, 0.5].$$

We can now define the change in circumference,

$$\Delta c = R\Delta\theta = 2\pi R\Delta\zeta\,,\; \Delta c \in [-\pi R, \pi R]$$

and the distance between the two points,

$$L_{ij} = \left\| \boldsymbol{r}_{ij} \right\| = \sqrt{\Delta z^2 + \Delta c^2}$$

Distance between a point and its downstream neighbour

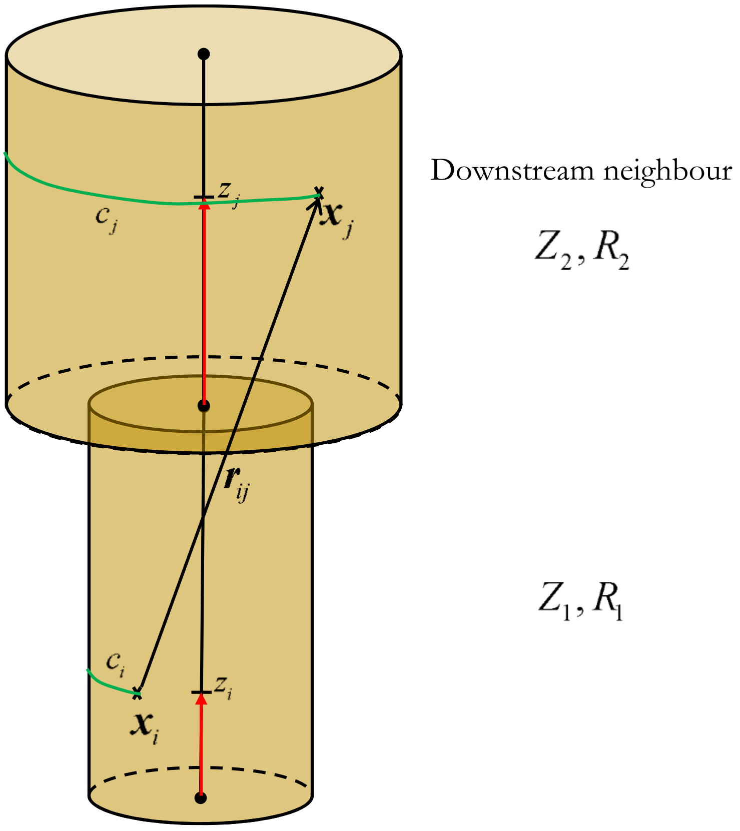

The point $\boldsymbol{x}_i$ resides within a cylinder with length Z_1 and radius R_1,

$$\{x_i\} = \begin{Bmatrix} z_i \\ c_i \end{Bmatrix} = \begin{Bmatrix} z_i \\ R_1\theta_i \end{Bmatrix} = \begin{Bmatrix} Z_1\xi_i \\ 2\pi R_1\zeta_i \end{Bmatrix}.$$

The point $\boldsymbol{x}_j$ resides in the upstream neighbour of this cylinder, with length Z_2 and radius R_2,

$$\{x_j\} = \begin{Bmatrix} z_j \\ c_j \end{Bmatrix} = \begin{Bmatrix} z_j \\ R_2\theta_j \end{Bmatrix} = \begin{Bmatrix} Z_2\xi_j \\ 2\pi R_2\zeta_j \end{Bmatrix}.$$

The distance between these two points is given by the magnitude of the vector $\boldsymbol{r}_{ij}$,

$$\boldsymbol{r}_{ij} = \boldsymbol{x}_j - \boldsymbol{x}_i = L_{ij}\hat{\boldsymbol{r}}_{ij},$$

$$\{r_{ij}\} = \begin{Bmatrix} \Delta z \\ \Delta c \end{Bmatrix}.$$

The longitudinal difference Δz can be calculated as,

$$\Delta z = (Z_1 - z_i) + z_j$$

$$= Z_1(1 - \xi_i) + Z_2\xi_j.$$

The circumferential difference Δc can be calculated as,

$$\Delta c = (R_2 - R_1)\theta_i + R_2\Delta\theta,$$

where $\Delta\theta$ has a similar range restriction as seen in the previous section,

$$\Delta\theta = \begin{cases} \theta_j - \theta_i, & \text{if } |\theta_j - \theta_i| \le \pi \\ (\theta_j - \theta_i) - 2\pi, & \text{if } (\theta_j - \theta_i) > \pi \\ (\theta_j - \theta_i) + 2\pi, & \text{if } (\theta_j - \theta_i) < -\pi \end{cases}.$$

We can the calculate the distance between the two points as

$$L_{ij} = \|\boldsymbol{r}_{ij}\| = \sqrt{\Delta z^2 + \Delta c^2}$$

Distance between a point and its upstream neighbour

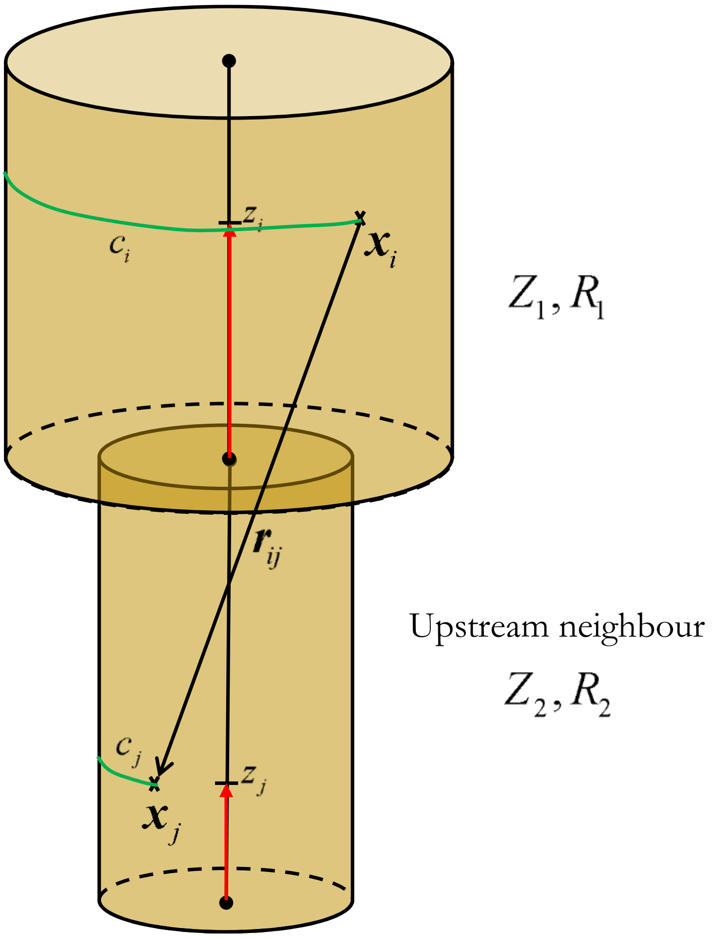

The point $\boldsymbol{x}_i$ resides within a cylinder with length Z_1 and radius R_1,

$$\{x_i\} = \begin{Bmatrix} z_i \\ c_i \end{Bmatrix} = \begin{Bmatrix} z_i \\ R_1\theta_i \end{Bmatrix} = \begin{Bmatrix} Z_1\xi_i \\ 2\pi R_1\zeta_i \end{Bmatrix}.$$

The point $\boldsymbol{x}_j$ resides in the downstream neighbour of this cylinder, with length Z_2 and radius R_2

$$\{x_j\} = \begin{Bmatrix} z_j \\ c_j \end{Bmatrix} = \begin{Bmatrix} z_j \\ R_2\theta_j \end{Bmatrix} = \begin{Bmatrix} Z_2\xi_j \\ 2\pi R_2\zeta_j \end{Bmatrix}.$$

The distance between these two points is given by the magnitude of the vector $\boldsymbol{r}_{ij}$,

$$\boldsymbol{r}_{ij} = \boldsymbol{x}_j - \boldsymbol{x}_i = L_{ij}\hat{\boldsymbol{r}}_{ij},$$

$$\{r_{ij}\} = \begin{Bmatrix} \Delta z \\ \Delta c \end{Bmatrix}.$$

The longitudinal difference Δz can be calculated as,

$$\Delta z = -z_i - \left(Z_2 - z_j\right)$$

$$= -Z_1\xi_i - Z_2\left(1-\xi_j\right).$$

The circumferential difference Δc can be calculated as,

$$\Delta c = \left(R_2 - R_1\right)\theta_i + R_2\Delta\theta ,$$

where $\Delta\theta$ has a similar range restriction as seen in the previous section,

$$\Delta\theta = \begin{cases} \theta_j - \theta_i, & \text{if } \left|\theta_j - \theta_i\right| \le \pi \\ \left(\theta_j - \theta_i\right) - 2\pi, & \text{if } \left(\theta_j - \theta_i\right) > \pi \\ \left(\theta_j - \theta_i\right) + 2\pi, & \text{if } \left(\theta_j - \theta_i\right) < -\pi \end{cases} .$$

We can the calculate the distance between the two points as

$$L_{ij} = \left\|\boldsymbol{r}_{ij}\right\| = \sqrt{\Delta z^2 + \Delta c^2} .$$

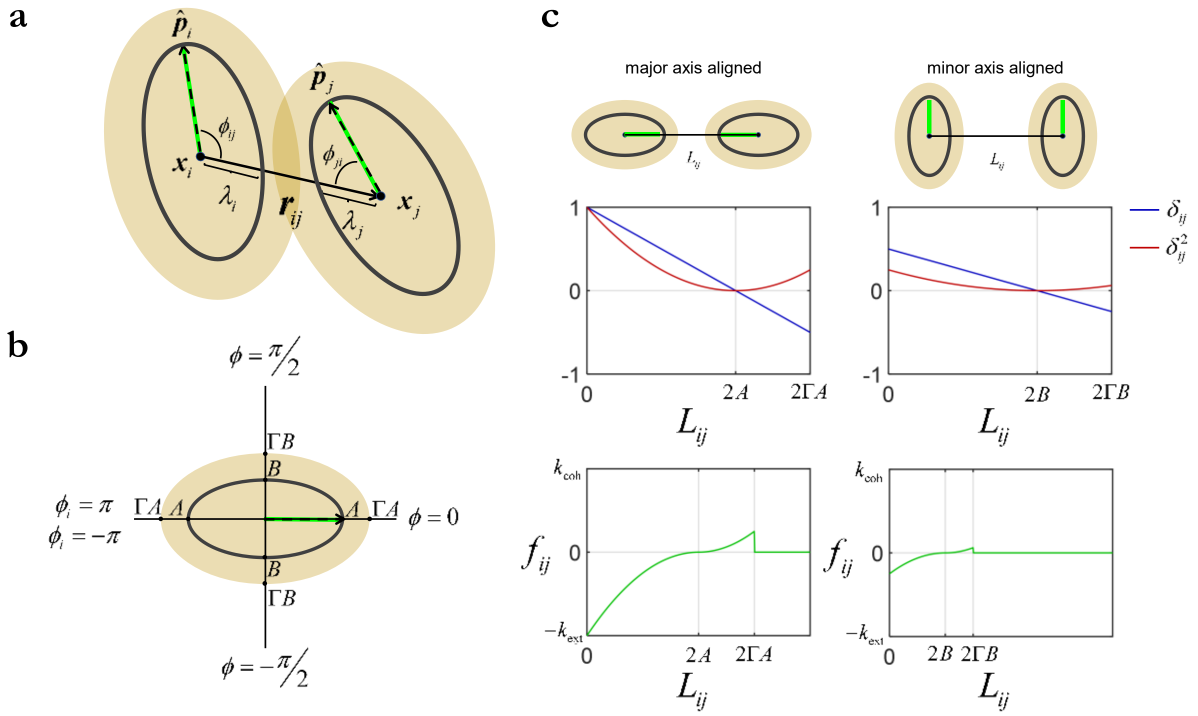

Supplementary Figure 12. Force transmission between agents based on overlap, δ. (A) The overlap between cell i and j is based on the length of the distance vector between them, $L_{ij} = \|r_{ij}\|$, the angles at which this vector intersects each agent, ϕ_{ij} and ϕ_{ji}, and the inner radius of intersection, λ_i and λ_j. (B) Each agent consists of an inner ellipse (semi-major axis A, semi-minor axis B) nested within an outer ellipse (semi-major axis ΓA, minor axis ΓB). The intersection angle is defined with respect to each cell's polarity vector $\hat{\boldsymbol{p}}$. (C) An example of overlap, both δ_{ij} and δ_{ij}^2, as well as transmission force f_{ij} for a pair of agents with major axis alignment (left) and minor axis alignment (right).
